# Adenylate Kinase 4 Promotes Inflammatory Gene Expression *via* Hif1α and AMPK in Macrophages

**DOI:** 10.3389/fimmu.2021.630318

**Published:** 2021-03-15

**Authors:** Wei-Yao Chin, Chi-Ying He, Tsun Wai Chow, Qi-You Yu, Liang-Chuan Lai, Shi-Chuen Miaw

**Affiliations:** ^1^Graduate Institute of Immunology, National Taiwan University College of Medicine, Taipei, Taiwan; ^2^Bioinformatics and Biostatistics Core, Center of Genomic and Precision Medicine, National Taiwan University, Taipei, Taiwan; ^3^College of Medicine, Graduate Institute of Physiology, National Taiwan University, Taipei, Taiwan

**Keywords:** AK4, classically activated macrophages (M1), HIF1α, AMPK, inflammation

## Abstract

Macrophages comprise the front line of defense against various pathogens. Classically activated macrophages (M1), induced by IFN-γ and LPS, highly express inflammatory cytokines and contribute to inflammatory processes. By contrast, alternatively activated macrophages (M2) are induced by IL-4 and IL-13, produce IL-10, and display anti-inflammatory activity. Adenylate kinase 4 (Ak4), an enzyme that transfers phosphate group among ATP/GTP, AMP, and ADP, is a key modulator of ATP and maintains the homeostasis of cellular nucleotides which is essential for cell functions. However, its role in regulating the function of macrophages is not fully understood. Here we report that Ak4 expression is induced in M1 but not M2 macrophages. Suppressing the expression of Ak4 in M1 macrophages with shRNA or siRNA enhances ATP production and decreases ROS production, bactericidal ability and glycolysis in M1 cells. Moreover, Ak4 regulates the expression of inflammation genes, including *Il1b, Il6, Tnfa, Nos2, Nox2*, and *Hif1a*, in M1 macrophages. We further demonstrate that Ak4 inhibits the activation of AMPK and forms a positive feedback loop with Hif1α to promote the expression of inflammation-related genes in M1 cells. Furthermore, RNA-seq analysis demonstrates that Ak4 also regulates other biological processes in addition to the expression of inflammation-related genes in M1 cells. Interestingly, Ak4 does not regulate M1/M2 polarization. Taken together, our study uncovers a potential mechanism linking energy consumption and inflammation in macrophages.

## Introduction

Macrophages comprise the front line of defense against pathogens (1). Two major functional subsets of macrophages have been identified (2, 3). Classically activated macrophages (M1) are induced by lipopolysaccharide (LPS)/interferon gamma (IFN-γ). They express high levels of inflammatory cytokines, such as interleukin-1β (IL-1β) (4), IL-6 (5), IL-12 (6), and tumor necrosis factor α (TNFα) (7) as well as nitric oxide synthase (iNOS) (8) that is responsible for nitric oxide (NO) production (9) and eradication of pathogens. By contrast, alternatively activated macrophages (M2) are induced by IL-4/IL-13. They produce cytokine IL-10 (10), and are crucial for anti-inflammatory responses, tissue repair and maintenance (11). Thus, macrophages can play both protective and pathogenic roles in many human diseases (12, 13).

Nine adenylate kinases (Ak1-9) with different organ distribution, subcellular localization and substrate specificity have been identified. Ak4 is located in the matrix of mitochondria. It is expressed mainly in mouse brain, heart, liver, stomach, kidney and ovary (14, 15). Ak4, encoded by gene *Ak4*, is a member of the adenylate kinase family. It is responsible for maintaining cellular nucleotide homeostasis by catalyzing the transfer of one phosphate group from one ATP or GTP to one AMP, resulting into two ADP or one ADP and one GDP (16, 17). Ak4 acts as a key regulator of ATP from different nutrient sources (18). Moreover, Ak4 plays a role in regulating the signaling of AMP-activated protein kinase (AMPK), a metabolic sensor that is activated through phosphorylation at Thr172 of its α-subunit by LKB1, TAK1, and Calcium/calmodulin-dependent protein kinase kinase II (CAMKK2). Phosphorylated AMPK can enhance mitochondria biogenesis to provoke ATP generation (18, 19).

A previous study has shown that Ak4 is involved in hypoxia tolerance. Its expression is enhanced under hypoxic conditions which induce hypoxia inducing factor 1α (Hif1α) (20). Furthermore, Ak4 is involved in resistance to anti-tumor drugs *via* regulating mitochondrial activity (19), and is a marker for metastasis and poor clinical outcome in lung cancer (21, 22).

Hif1α, encoded by the *Hif1a* gene, is a member of the basic helix-loop-helix PAS superfamily (23, 24). Hif1α protein comprises four functional regions, basic helix-loop-helix domain, PAS domain, stability determining domain, and trans-activating domain (25). Hif1α plays a crucial role for cellular and developmental response to low oxygen concentration (hypoxia) (26, 27). Moreover, Hif1α is involved in tumor growth, survival and metastasis (28–30). Metabolic state of an M1-polarized macrophage favors glycolysis (31). As a metabolic regulator of glycolysis in macrophages, Hif1α induces M1 polarization and activation that promotes inflammatory gene expression, bacterial killing and cell migration (32, 33). Here, we demonstrate that Ak4 is highly expressed in M1 subset compared to M0 and M2 subsets. It not only maintains ATP homeostasis, ROS production and glycolysis but also has a broad impact on the transcriptome of M1 cells. More importantly, it promotes the expression of inflammation genes through a positive feedback loop formed with Hif1α and down-regulates AMPK phosphorylation. Moreover, we show that Ak4 does not regulate M1/M2 polarization. Our work reveals a novel association between metabolism and inflammation.

## Materials and Methods

### Mice

The 6–8-week-old female C57BL/6 mice were purchased from National Laboratory Animal Center. All animals were housed in Laboratory Animal Center of National Taiwan University College of Medicine under specific pathogen-free (SPF) condition. Water and food were provided sufficiently daily, and all mice were sacrificed by CO_2_. This study was carried out in strict accordance with the recommendations in the Guide for the Care and Use of Laboratory Animals of the National Institutes of Health (NIH). The protocol was approved by the Institutional Animal Care and Use Committee of National Taiwan University College of Medicine (Permit Number: 20190119).

### Macrophages Culture and M1/M2 Polarization

Bone marrow cells were isolated from femur and tibia bones of mice with incomplete DMEM (Dulbecco's modification of Eagle's medium, Gibco, Darmstadt, Germany) by using 23G needles, and centrifuged at 4°C with 800 × g for 5 min. Red blood cells (RBC) were lysed by 900 μL RBC lysis buffer (10 mM Tris-HCl and 0.83% NH_4_Cl in ddH_2_O, pH 7.2) for 3 min on ice, and 100 μL 10X Dulbecco's phosphate-buffered saline (DPBS, Gibco) was added to cells. Cells were then centrifuged at 4°C with 800 × g for 5 min, and re-suspended with complete DMEM containing 10% fetal bovine serum (FBS, Corning, New York, United States), 1X penicillin/streptomycin (HyClone), 1X L-Glutamine (HyClone), 1X NEAA (non-essential amino acid, HyClone), 1X sodium pyruvate (HyClone), and 5 μM 2-Mercaptoethanol (2-ME, Thermo, Massachusetts, U.S.). 5 × 10^6^ cells or 2 × 10^6^ were seeded in 10-cm^2^ petri dishes (α-Plus) or 6-well plates, respectively, at 37°C in 5% CO_2_ incubator for 7 days. Cells were cultured in 20% L929 cell supernatant in complete DMEM. After culturing in 20% L929 cell supernatant for 7 days, bone marrow-derived macrophages (BMDMs) were collected by trypsinization and further seeded in 12-well and 24-well plates (Thermo) at a density of 1 × 10^6^/mL and 5 × 10^5^/mL per well for Western blot and real-time PCR (qPCR) analysis, respectively. For macrophages polarization, BMDMs were treated with LPS (1,000 ng/mL, O111:B4 *E. coli*) and IFN-γ (20 ng/mL, Peprotech, New Jersey, USA) or IL-4 (20 ng/mL, Peprotech) and IL-13 (20 ng/mL, Peprotech) for M1 and M2 polarization, respectively. To isolate peritoneal macrophages, C57BL/6 mice were injected with 1 mL of sterile 3.8% thioglycollate medium by intraperitoneal injection. On day 4, mice were euthanized and peritoneal cells were collected *via* peritoneal lavage with 5 ml sterile PBS. Cells were then spun down for 5 min at 800 × g, resuspended in complete RPMI and seeded 1 × 10^6^ cells/well in 12-well plate. After 24 h, the non-adherent cells were removed. Adherent cells were collected and stained with anti-CD11b and anti-F4/80 to confirm the macrophage population. M1/M2 polarization was defined by flow cytometry and qPCR. M1 dominant expression markers were CD86, *Nos2* and *Tnfa*. M2 dominant expression markers were CD206, *Arg1* and *Egr2* (Supplementary Figure 1).

### DMOG, Metformin or A-769662 Treatment in BMDMs

To stabilize Hif1α in M1 at normoxia condition (34), BMDMs were treated with 0.2 mM DMOG (Merck, Germany), a PHD inhibitor, and cell lysate was collected after DMOG treatment for 24 h. In some experiments, metformin and A-769662 (Merck), which enhance AMPK activation, were added to BMDMs to a final concentration of 10 and 20 μM, respectively, 30 min before LPS stimulation. On 24 h after treatment with metformin or A-769662, cell lysate was collected.

### Lentiviral Plasmid Construction for Ak4 Overexpression

pcDNA3.1 which carried mouse Ak4 open reading frame (ORF) with flag tag (CloneID: OMu1602) was purchased from Genscript. Ak4 ORF with flag tag was amplified by PCR, and then purified by gel extraction kit (Geneaid, Taiwan, ROC). PCR product was constructed into pWPI lentiviral vector at SwaI site by Genbuilder cloning kit (Genscript, Piscataway Township, USA). The final construct was confirmed by DNA sequencing. The primer sequences are listed in Table 1.

**Table 1:** The primer list.

**Gene**	**Sequence (5^**′**^->3^**′**^) (F)**	**Sequence (5^**′**^->3^**′**^) (R)**
*Actb*	TGT ATG AAG GCT TTG GTC TCC CT	AGG TGT GCA CTT TTA TTG GTC TCA A
*Il1b*	TAC GGA CCC CAA AAG ATG A	TGC TGC TGC GAG ATT TGA AG
*Il6*	CCG GAG AGG AGA CTT CAG AG	TCC ACG ATT TCC CAG AGA AC
*Tnfa*	CAT CTT CTC AAA ATT CGA GTG ACA A	TGG GAG TAG ACA AGG TAC AAC CC
*Ccl2*	TTAAAAACCTGGATCGGAACCAA	GCATTAGCTTCAGATTTACGGGT
*Cxcl3*	CATCCAGAGCTTGACGGTCAC	GCCGCTCTTCAGTATCTTCTTG
*Il10*	CAT ACT GCT AAC CGA CTC CTT	CTC CAC TGC CTT GCT CTT AT
*Hif1a*	AGC CCT AGA TGG CTT TGT GA	TAT CGA GGC TGT GTC GAC TG
*Nos2*	TGC ATG GAC CAG TAT AAG GCA AGC	GCT TCT GGT CGA TGT CAT GAG CAA
*Arg1*	CAG AAG AAT GGA AGA GTC AG	CAG ATA TGC AGG GAG TCA CC
*Egr2*	CCT CCA CTC ACG CCA CTC TC	CAC CAC CTC CAC TTG CTC CTG
*Chi3*	TCA CAG GTC TGG CAA TTC TTC TG	TTT GTC CTT AGG AGG GCT TCC TCG
*Retnla*	GGT CCC AGT GCA TAT GGA TGA GAC CAT AGA	CAC CTC TTC ACT CGA GGG ACA GTT GGC AGC
*IRF4*	TCC GAC AGT GGT TGA TCG AC	CCT CAC GAT TGT AGT CCT GCT T
*IRF7*	ACA GGG CGT TTT ATC TTG CG	TCC AAG CTC CCG GCT AAG T
*Socs1*	ACT TCT GGC TGG AGA CCT CA	ACA AGC TGC TAC AAC CAG GG
*Socs3*	CCT TCA GCT CCA AAA GCG AG	GCT CTC CTG CAG CTT GCG
*Ak1*	GTC GGC TAT CAT GGA GAA GG	AGT CGT TGG GTC ATG GTC TC
*Ak2*	GGG AAA CTG GTG AGT GAC GA	ATC AAG CAT TTC AGC CTG CC
*Ak3*	GCA TTG ATG ACC TGA CCG GA	CAG AGA ATG TTT CCA ACA CCC C
*Ak4*	AAA GGA TCG CCC AGA ACT TT	TCG GGA ATC CAT CTA ACA GC
*Ak5*	GGC TCC GAT GGA TTC AAG TG	CAT GAA GCC TCC CTC TGT GT
*Ak6*	GCA GTT ATA CGA CGG CTA CGA	TAT GAA ACC AGC GTT CGG GG
*Ak7*	AGG CTT CGT GGA GAA CAT CA	ATC GCC TCC AGT TTG GCT AT
*Ak8*	GCA ACA AGA TTG CAT CCA GA	CAG GGT CTG TCC TTC TCA GC
*Ak9*	TCC TGA TAA TGA AGC CGA GGA G	CCC TCG GGT GTA CTC AGG T
Ak4 construction	GAG GAA TTT CGA CAT TTG CCA CCA TGG CTT CCA A	CGA TAC CGT CGA GAT TAT TAT CAC TTA TCG TCG T
Ak4-pWPI sequencing	GGC CAG CTT GGC ACT TGA TG	GAA TTC CTG CAG CCC GTA GT

*Sequences of primers used for Quantitative Real-time PCR were listed*.

### Lentiviral Production and Transduction

Lentiviral constructs carry GFP and Ak4 (TRCN0000345103; 5′CTGTGATGTGGACCTAGTAA T3′) or Hif1α (TRCN0000232222; 5′TGGATAGCGATATGGTCAATG3′) shRNA were purchased from Academia Sinica, ROC. The plasmids amplifications were followed by Academia Sinica's protocol. Plasmids psPAX2, pMD2.G and target plasmid including pWPI lentiviral plasmid carry Ak4 were co-transfected into HEK293T cells with Lipofectamin 3000 (Invitrogen). The supernatant was condensed with 0.4 M NaCl and 8.5% PEG6000 at 4°C overnight (O/N). The viruses were spun down by 7,000 × g at 4°C for 20 min, re-suspended in dPBS containing 2%FBS and HBSS (Gibco), and stored in −80°C. Lentiviral transduction was conducted in serum free medium containing 20% L929 supernatant with 8 μg/ml polybrene at day 3 of BMDM culture (MOI = 30). The medium containing lentivirus and polybrene was replaced with complete medium containing 20% L929 supernatant at day 4. The infected BMDMs were polarized into M1 or M2 macrophages. GFP^+^ cells were sorted out for RNA-Sequencing, qPCR, ATP, and ADP/ATP assay after M1 polarization for 24 h.

### siRNA Transfection

BMDMs were collected after culture bone marrow cells in 20% L929 supernatant for 7 days. Cells were washed twice with PBS and rested in complete medium for 3 h. Ak4 siRNA or AMPKα1/2 siRNA in 50 nM was transfected into BMDMs with Lipofectamin 3000 (thermo) for 24 h before LPS/IFN-γ stimulation. To knockdown Ak4 in peritoneal macrophage, after removing the non-adherent cells, 50 nM Ak4 siRNA were transfected into peritoneal macrophages with Lipofectamin 3,000 for 24 h before LPS/IFN-γ stimulation.

### Metabolic Extracellular Flux Analysis

For real-time analysis of oxygen consumption rate (OCR) and extracellular acidification rate (ECAR), 10^5^ BMDMs were seeded in XF-96 cell culture plates, and stimulated with LPS/IFN-γ for 24 h. OCR and ECAR, which represent oxidative phosphorylation and glycolysis ability, respectively, were analyzed on XF-96 analyzer. For OCR analysis, 1 μM oligomycin, 2 μM fluoro-carbonyl cyanide phenylhydrazone (FCCP), and 100 nM rotenone plus 1 μM antimycin A (Rot/AA) were injected in sequence. For ECAR analysis, 60 mM glucose, 1 μM oligomycin, and 600 mM 2-deoxy-D-glucose (2-DG) were injected in sequence.

### Quantitative Real-Time PCR (qPCR)

Total RNA was extracted from BMDMs using TRIzol reagent. RNA isolation kit, direct-zol RNA miniPrep (Zymo Research, Irvine, California, USA), was used according to the manufacturer's instruction. RNA was quantified with DS-II^+^ spectrophotometer (DeNoyix). The complementary DNA (cDNA) was generated using MMLV high performance reverse transcriptase (MMLV HP RT, Epicenter Biotechnologies, California, USA). Real-time PCR was performed on a PikoReal 96 Real-Time PCR System (Thermo Scientific) using SYBR green mixture (Bioline, London, UK). Relative expression of the target gene was normalized to *Actb* and calculated as 2^–(Ct target–Ct β-actin)^. The Ct represents the threshold cycle for each target or reference gene determined by Thermo PikoReal Software 2.1. The relative target gene expression was calculated by using the 2^−Δ*ΔCt*^ method. Sequences of primers used for quantitative real-time PCR were listed (Table 1).

### Flow Cytometry

In brief, 5 × 10^5^ BMDMs were seeded on 24-well plate after 7-days culture. For measuring cytosol and mitochondria ROS, CellRox and MitoSox probes were used, respectively, as manufacturer's protocol (Thermo Scientific). In brief, BMDMs were treated with LPS (1,000 ng/mL) and IFN-γ (20 ng/mL) for M1 polarization. Twenty-four hours later, M1 cells were washed with PBS two times and stained with CellRox or MitoSox diluted in staining buffer at 37°C for 15 min before analyzed by flow cytometry. To sort GFP^+^ cells, propidium iodide (PI, Biolegend) staining was used to identify viable cells. Samples were analyzed on LSR Fortessa (BD Biosciences, Franklin Lakes, New Jersey, USA) or sorted on FACSAriaIII and data were analyzed using FlowJo software.

### Western Blot Analysis

Cells were lysed with RIPA (25 mM Tris-HCl pH7.6, 150 mM NaCl, 1% NP-40, 1% sodium deoxycholate and 0.1% SDS) on ice for 30 min, and centrifuged at 4°C with 20,000 × g for 20 min for the supernatant. Total protein concentrations were measured with Protein Assay Dye (Thermo scientific). The samples were separated on 8 or 12% SDS-PAGE, and transferred to 0.22 μm PVDF membranes. The membranes were blocked with 5% skim milk, and probed with primary antibodies p-AMPK, AMPK (Cell signaling technology), iNOS, HIF1α, and Ak4 (GeneTex, Taiwan, ROC) at 4°C shaking O/N. The membranes were washed with TBST, and incubated in goat anti-rabbit secondary antibody with HRP conjugated (Abcam) for 1 h at room temperature. Membranes were soaked in enhanced chemiluminescent reagent (GE healthcare), exposed to film or iBright 1500 Western Blot Imaging Systems. The expression of protein was quantified by ImageJ or app in Thermo fisher website. α-Tubulin, β-Actin, or COX IV (Abcam) as an internal control was re-probed to PVDF membranes, after stripping.

### Phagocytosis and Killing Assay

Five hundred thousand BMDMs were seeded on 24-well plates the day prior to the experiment. *E coli* were added to BMDMs (MOI = 2 or 10) after the culture medium were changed to P/S-free DMEM. The plates were incubated at 37°C for 1 h. BMDMs were washed with PBS twice, changed to 100 μg/ml gentamicin-containing DMEM, and incubated at 37°C for 1 h. BMDMs were then lysed with 0.1% tritonX-100 and plated for phagocytosis assay. BMDMs were incubated at 37°C for another 4 h, then lysed with 0.1% tritonX-100 and plated for killing assay. Colony forming unit (CFU) was counted after 24 h incubation.

### Mitochondria Extraction

M1 cells were lysed with extraction buffer (1 M sucrose, 1 mM EGTA, 5 mM Tris-HCl pH 7.4). The process of Mitochondria extraction was performed following a published protocol (35). In brief, BMDMs (5 × 10^7^) were lysed with 3 ml extraction buffer (1 M sucrose, 1 mM EGTA, 5 mM Tris-HCl pH 7.4) on ice for 30 min. Cell lysate was centrifuged with 300 × g for 10 min. The supernatant was transferred into a new tube, and centrifuged with 7,000 × g for 10 min. The pellet was washed with 1 ml extraction buffer twice. Protein lysate (50 μg) was used for Western blot.

### Enzyme-Linked Immunosorbent Assay

To measure IL-1β, IL-6, and TNF-α production, culture supernatants from BMDMs and peritoneal macrophages in 12-well plates were collected. Mouse IL-1β, IL-6, and TNF-α ELISA kit (Biolegend) was used following the manufacturer's instructions.

### ATP and ADP/ATP Assay

The amount of ATP and ADP/ATP were measured by ApoSENSOR Cell Viability Assay Kit and ApoSENSOR ADP/ATP Ratio Bioluminescent Assay Kit (BioVision, California, USA) following the manufacturer's instructions. In brief, to measure intracellular ATP, 5 × 10^4^ GFP^+^ M1 cells were lysed with 250 μl lysis buffer on ice for 5 min. ATP monitor enzyme and lysis buffer were pre-mixed and the background luminescence value was designated as A. Cell lysate (50 μl) was added to the pre-mixed well, then the luminescence reading was designated as B. ADP converting enzyme was added to the well, then the luminescence reading was designated as C. The value of intracellular ATP is (B-A). The value of intracellular ADP/ATP is (C-B)/(B-A). The tests were done in duplicates.

### Next-Generation Sequencing and Analysis of Ak4-Regulated Genes in M1 Cells

Total RNA was extracted from M1 cells and digested to 180 bp fragments after removing rRNAs with a Ribo-Zero Gold kit (Illumina, San Diego, USA). A sequencing library was created by reverse-transcription of RNA fragments to cDNA, and ligating specific adapters at both ends of these cDNA fragments. Next, the sequencing libraries were loaded to the surface of flow cells, and amplified into clonal clusters though bridge amplification. All of the clusters on the flow cell were sequenced by NextSeq 500 (Illumina). FastQC (v0.11.8) program (http://www.bioinformatics. babraham.ac.uk/projects/fastqc/) was used to examine the quality of sequencing reads, and HISAT2 (v2.1.0) was used to align sequencing reads to mouse genome references (GRCm38) (36). The output SAM files were normalized and quantified with R packages Rsubread (v 2.0.0) (37) and edgeR (v 3.28.0) (38) from Bioconductor. A robust normalization approach, the trimmed mean of M value (39), was applied and log_2_-transformation was used to obtain the final expression values. In the differential gene expression analyses, Student's *t*-test (*P* < 0.01) and 2-fold changes were used to identify differentially expressed (DE) genes between *Ak4* shRNA knockdown samples and scramble samples. The functional and pathway analyses for DE genes were further analyzed by Ingenuity Pathway Analysis (QIAGEN Inc., https://www.qiagenbioinformatics.com/products/ingenuity-pathway-analysis). All of the data have been deposited in Gene Expression Omnibus (GEO, GSE143302).

### Statistical Analysis

Statistical analyses were performed with unpaired, two-tailed Student's *t*-test or one-way ANOVA for multiple comparisons. A value of *P* < 0.05 was considered statistically significant.

## Results

### Ak4 Is Preferentially Expressed in M1 Macrophages

**Figure 1:**
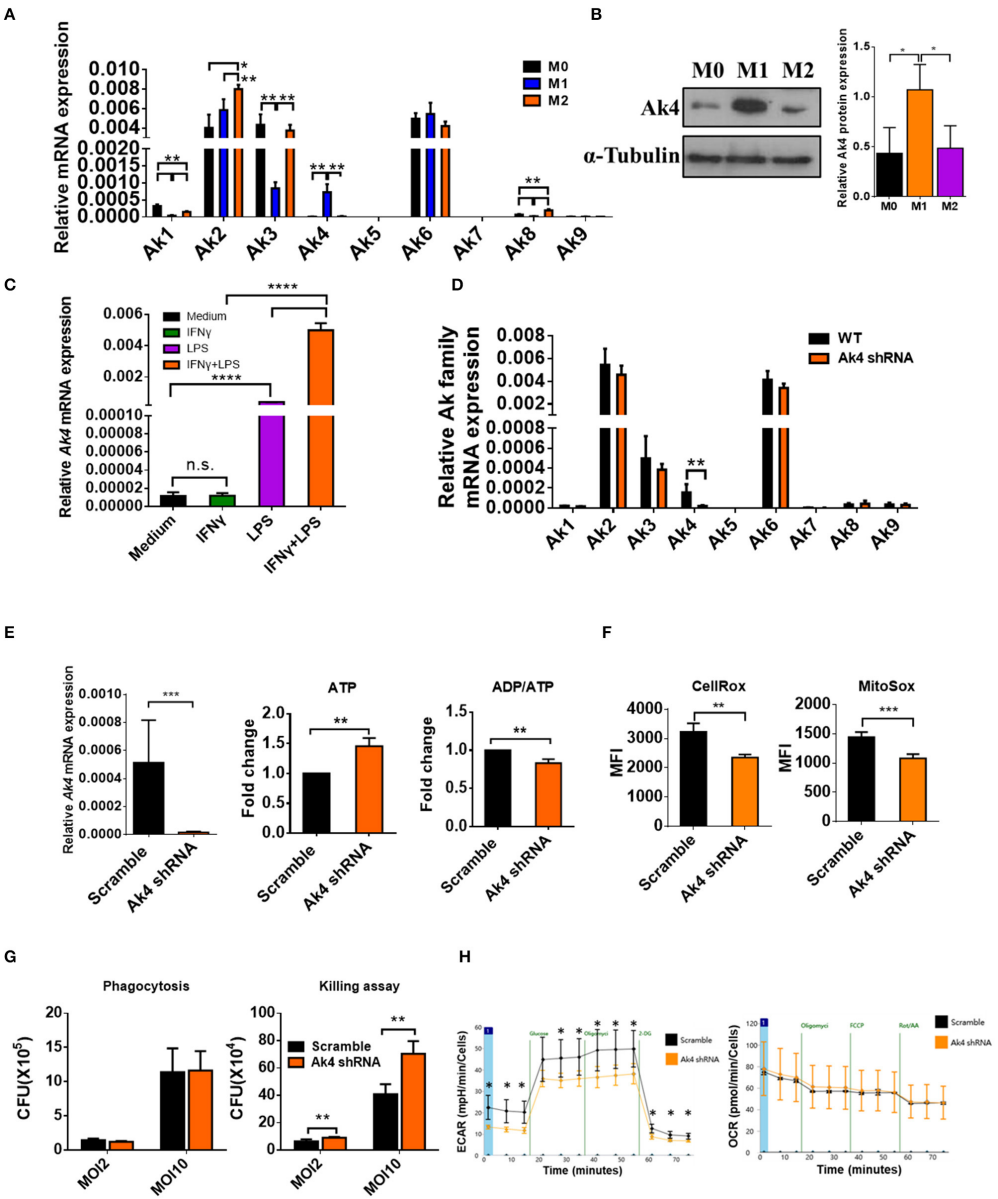
Ak4 is highly expressed in M1 macrophages and it maintains ATP homeostasis, ROS production, bactericidal ability, and glycolysis. BMDMs (M0) were treated with 1,000 ng/mL LPS and 20 ng/mL IFN-γ (for M1 polarization) or 20 ng/mL IL-4 and 20 ng/mL IL-13 (for M2 polarization) for 24 h. **(A)** Ak1-9 mRNA expressions were measured by qPCR (*n* = 3). The mRNA expressions were normalized against *Actb*. **(B)** Ak4 protein expressions were analyzed by Western blotting. Relative protein expressions were normalized against α-Tubulin (*n* = 3). **(C)** BMDMs were treated with IFN-γ, LPS, LPS+IFN-γ or medium only for 24 h. Ak4 mRNA expressions were measured by qPCR (*n* = 4). mRNA expressions were normalized against *Actb*. **(D)** Relative expressions of Ak1-9 mRNA in scramble shRNA- and Ak4 shRNA-treated M1 macrophages were analyzed by qPCR (*n* = 3). **(E)** Virus carrying scramble shRNA or Ak4 shRNA and GFP were transduced into BMDMs. After LPS/IFN-γ stimulation for 24 h, GFP^+^ M1 macrophages were sorted for expression of relative Ak4 mRNA, ATP level and ADP/ATP ratio. Relative Ak4 mRNA expressions were normalized against *Actb, n* = 3. ATP level and ADP/ATP ratio in scramble shRNA- and Ak4 shRNA-treated M1 cells were measured, *n* = 7. **(F)** CellRox and MitoSox were used to stain scramble shRNA- and Ak4 shRNA-treated M1 cells and cells were analyzed by flow cytometry (*n* = 3). **(G)** Scramble and Ak4 shRNA BMDMs were infected with *E. coli* with MOI 2 or 10. For phagocytosis and killing assay, cells were lysed with triton-X 100 and plated on LB agar plates. CFU was counted after culture for 24 h (*n* = 3). **(H)** ECAR and OCR were measured in Ak4 shRNA- and scramble shRNA-treated M1 cells by XF-96 analyzer (*n* = 3). *P < 0.05; **P < 0.01; ***P < 0.001; ****P < 0.0001 determined by one-way ANOVA **(A–C)** or unpaired, two-tailed Student's *t*-test **(D–H)** with mean ± SD.

To examine the expression of Ak family members in functional subsets of macrophages, we differentiated total bone marrow cells into macrophages (bone marrow-derived macrophage, BMDM) in 20% L929 conditioned medium for 7 days *in vitro*. BMDMs were subsequently left unstimulated (M0) or activated with LPS+IFN-γ (M1) or IL-4+IL-13 (M2) for 24 h. The mRNA expression of Ak family members among M0, M1 and M2 were analyzed by qPCR. While the expressions of Ak5, Ak7, and Ak9 were too low to be detected, that of Ak6 was comparable among M0, M1, and M2. Interestingly, the levels of Ak1, Ak2, Ak3, and Ak8 were higher in M2 than M1 cells. Notably, Ak4 was expressed almost exclusively in M1 cells and it located in mitochondria (Figure 1A; Supplementary Figure 2). Consistently, we found that Ak4 protein level in M1 macrophages was about two to three folds of that of M0 and M2 macrophages (Figure 1B). Moreover, we found that Ak4 expression was mainly induced by LPS stimulation. LPS and IFN-γ co-treatment further enhanced Ak4 expression in BMDMs (Figure 1C). These results suggest that Ak4 plays an important role in M1 macrophages. Therefore, we focus our study on characterizing the role of Ak4 in M1 macrophages.

### Ak4 Is Critical for Maintaining ATP Homeostasis, ROS Production, Bactericidal Ability and Glycolysis in M1 Macrophages

To investigate the role of Ak4 in M1 macrophages, BMDMs were transduced with Ak4 shRNA or scramble lentivirus for 5 days followed by stimulation with LPS and IFN-γ for 24 h. Transduced cells were sorted for *in vitro* analysis. The transcript level of Ak4 was reduced by more than 80%, whereas the expression of other Aks was not affected by the Ak4 shRNA (Figure 1D). In addition, cellular ATP level was elevated and ADP/ATP ratio was reduced by Ak4 shRNA and Ak4 siRNA (Figure 1E; Supplementary Figure 3A). Moreover, cytosol and mitochondrial ROS production were decreased in Ak4 shRNA-treated M1 cells (Figure 1F). Phagocytosis and bacterial clearance are two major functions of macrophages. Killing of bacteria is mediated by ROS and NO (40, 41). We infected Ak4 shRNA- and scramble shRNA-treated BMDMs with *E coli*. While bacteria uptake was not altered by either scramble or Ak4 shRNA, bacteria clearance ability was reduced in Ak4 shRNA-treated BMDMs at both MOI 2 and 10 compared to scramble shRNA-treated BMDMs (Figure 1G). Interestingly, both glycolysis and glycolytic capacity decreased in Ak4 shRNA-treated M1 cells. By contrast, the oxygen consumption rate (OCR) was not affected by the Ak4 shRNA (Figure 1H). These data indicate that Ak4 plays a non-redundant role in maintaining the homeostasis of ATP and ROS, sustaining bactericidal ability, and promoting glycolysis in M1 macrophages.

### Ak4 Regulates the Expression of Inflammation Genes in M1 Macrophages

**Figure 2:**
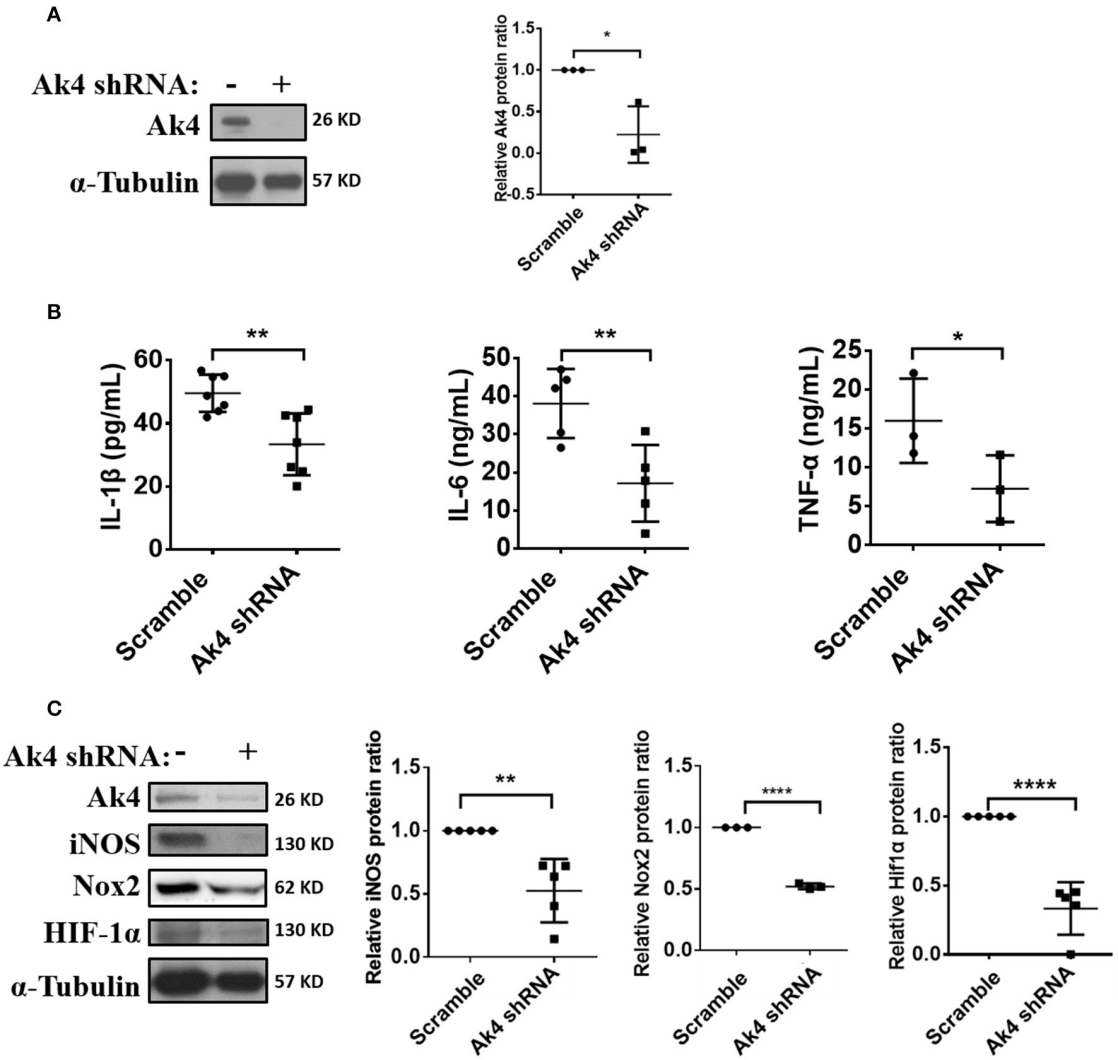
Expressions of inflammation-related protein in Ak4 shRNA-treated M1 macrophages. Pro-inflammatory cytokines IL-1β, IL-6, TNF-α, Nox2, and iNOS expression in Ak4 shRNA- and scramble shRNA-treated M1 macrophages were analyzed by ELISA or Western blotting. Relative protein expressions were normalized against α-Tubulin. **(A)** Relative protein expressions of Ak4 in Ak4 shRNA- and scramble shRNA-treated M1 macrophages (*n* = 3). **(B)** Expressions of IL-1β (*n* = 7), IL-6 (*n* = 5), and TNF-α (*n* = 3) in Ak4 shRNA- and scramble shRNA-treated M1 macrophages were measured by ELISA. **(C)** Expressions of iNOS (*n* = 5), Nox2 (*n* = 3), and Hif1α (*n* = 5) in Ak4 shRNA- and scramble shRNA-treated M1 macrophages were analyzed by Western blotting. *P < 0.05; **P < 0.01; ****P < 0.0001 determined by unpaired, two-tailed Student's *t*-test with mean ± SD.

IL-1β, IL-6, TNF-α, iNOS (encoded by *Nos2*), and Nox2 (encoded by *Nox2*) promote inflammatory responses to eradicate pathogens in M1 macrophage. Ak4 protein level was reduced in Ak4-silenced M1 cells (Figure 2A). Interestingly, the expressions of *Il1b, Tnfa*, and *Il6* were also down-regulated in cells treated with Ak4 shRNA and siRNA (Supplementary Figures 3B,C,F,G). Silencing Ak4 by siRNA in LPS/IFN-γ-treated thioglycollate-elicited peritoneal macrophages also reduced *Il1b, Tnfa*, and *Il6* transcripts (Supplementary Figure 3I). ELISA assay confirmed that silencing Ak4 reduced the protein levels of IL-1β, IL-6, and TNFα (Figure 2B; Supplementary Figure 3J). Moreover, *Hif1a* and *Nos2* transcripts as well as iNOS, Nox2, and Hif1α proteins were reduced by Ak4 shRNA and Ak4 siRNA treatments (Supplementary Figures 3D,E,H; Figure 2C). The data suggest that Ak4 positively regulates the expression of pro-inflammatory cytokines, iNOS, Nox2, and Hif1α in M1 cells.

### Ak4 and Hif1α Form a Positive Feedback Loop in Promoting the Expression of Inflammation Genes in M1 Macrophages

**Figure 3:**
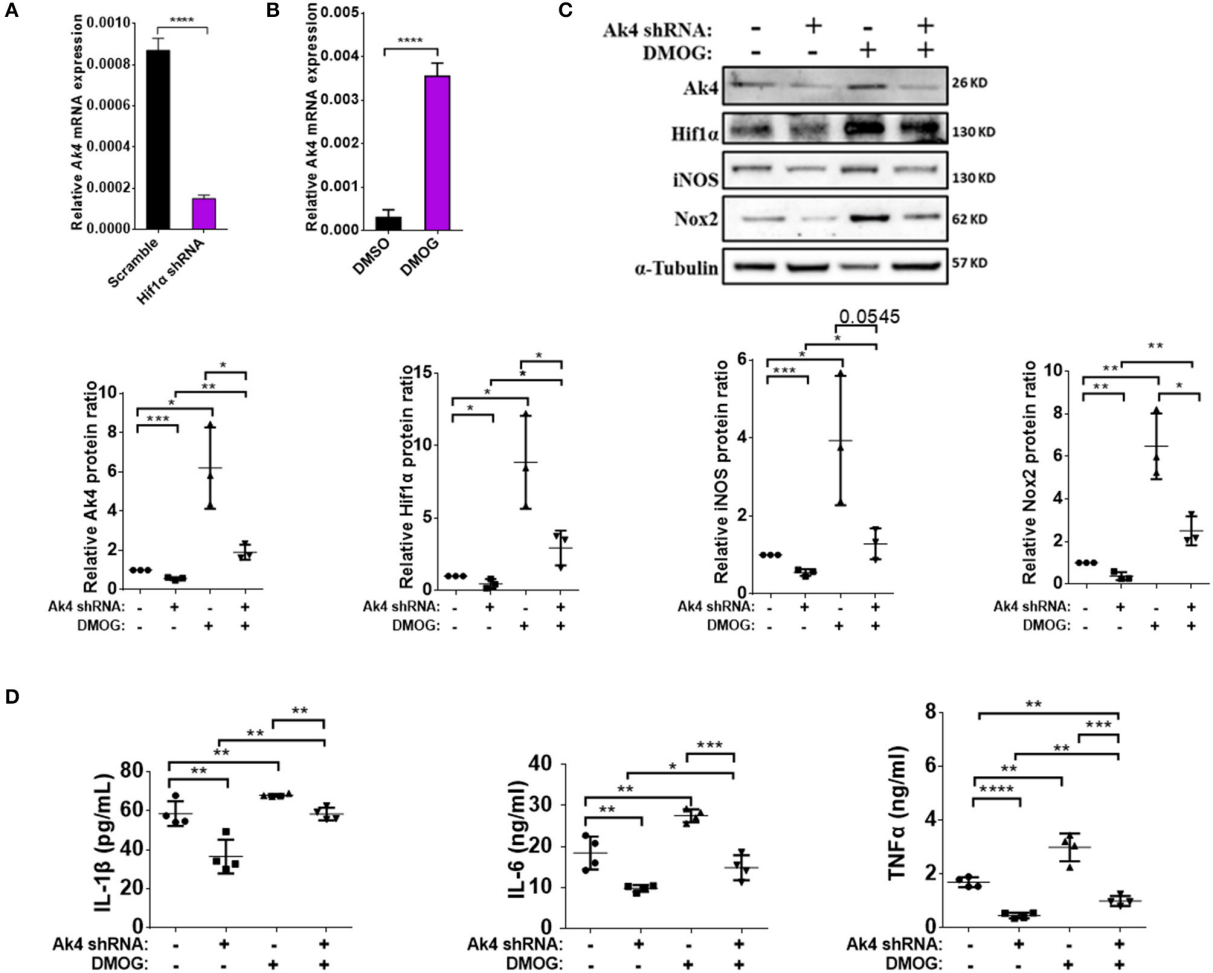
Pro-inflammatory cytokines IL-1β, IL-6, TNF-α, Nox2, and iNOS are downstream targets of Ak4-Hif1α feedback loop in M1 macrophages. Expressions of Ak4 mRNA in Hif1α shRNA- and scramble shRNA- or DMOG-treated M1 macrophages were analyzed by quantitative real-time PCR. Expression of Ak4, Hif1α, iNOS, and Nox2 protein and pro-inflammatory cytokines IL-1β, IL-6, and TNF-α in Ak4 shRNA- and scramble shRNA-treated M1 macrophages with or without 0.2 mM DMOG treatment were analyzed by Western blotting and ELISA, respectively. Relative mRNA expressions were normalized against *Actb*. Relative protein expressions were normalized against α-Tubulin. **(A,B)** Relative mRNA expression of Ak4 in **(A)** Hif1α shRNA- and scramble shRNA-treated M1 macrophages (*n* = 7) or **(B)** DMOG-treated M1 macrophages (*n* = 8). **(C)** Expressions of Ak4, Hif1α, iNOS, and Nox2 were analyzed by Western blotting (*n* = 3). **(D)** Productions of cytokines IL-1β, IL-6, and TNF-α were measured by ELISA (*n* = 4). *P < 0.05; **P < 0.01; ***P < 0.001; ****P < 0.0001 determined by unpaired, two-tailed Student's *t*-test **(A,B)** or one-way ANOVA **(C,D)** with mean ± SD.

Hif1α reportedly up-regulates IL-1β and iNOS (42). In agreement with this report, we found that suppressing the expression of Hif1α with shRNA resulted in downregulation of IL-1β, IL-6, TNF-α, and iNOS (Supplementary Figures 4A–C). Reversely, treating M1 cells with dimethyloxalylglycine (DMOG), which is known to stabilize Hif1α protein even in normoxia conditions, had an opposite effect (Supplementary Figures 4D–F). Surprisingly, the expressions of *Ak4* was down-regulated in Hif1α shRNA-treated M1 macrophages and up-regulated in M1 macrophages treated with DMOG (Figures 3A,B). These observations are consistent with published data showing that the expression of Ak4 increased under hypoxia environment (19) and strongly suggest that Ak4 and Hif1α form a positive feedback loop in M1 cells.

To investigate the role of the Ak4-Hif1α feedback loop in regulating the expression of inflammation genes in M1 cells, we transduced BMDMs with Ak4 shRNA or scramble shRNA for 5 days, then treated the cells with DMOG for 4 h before stimulation with LPS and IFN-γ. We found that DMOG restored the expression of Ak4, Hif1α, iNOS, Nox2, IL-1β, IL-6, and TNF-α in Ak4 shRNA-treated M1 cells (Figures 3C,D). Reversely, the effect of DMOG on the expression of Hif1α was much blunted by Ak4 shRNA.

### Ak4 Inhibits the Activation of AMPK to Promote Inflammation Gene Expressions in M1 Macrophages

**Figure 4:**
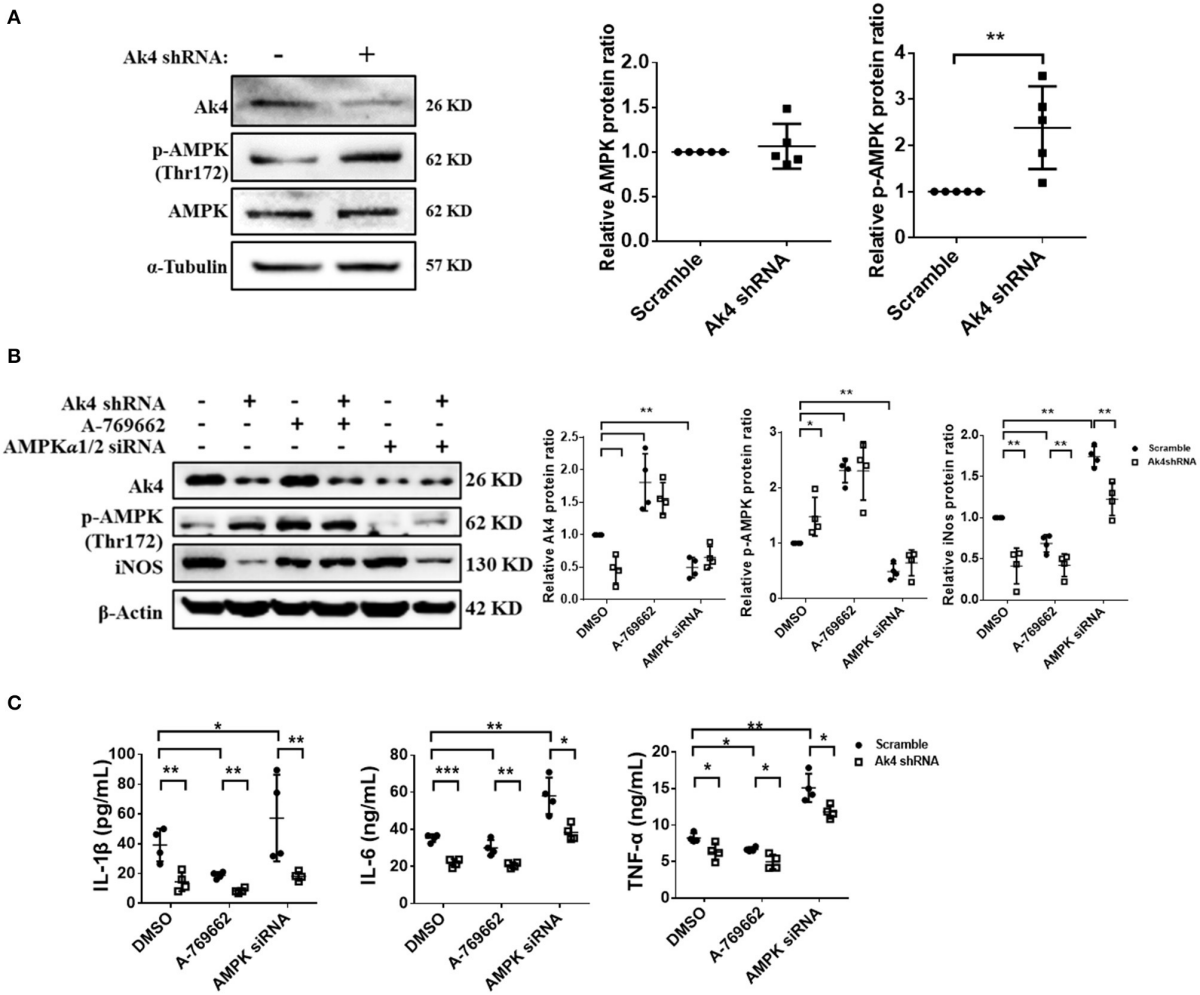
Ak4 inhibits the activation of AMPK to promote inflammation gene expressions in M1 macrophages. Expressions of Ak4, p-AMPK, AMPK in scramble shRNA- and Ak4 shRNA-treated M1 macrophages were analyzed by Western blotting. Relative protein expressions were normalized against β-actin or α-Tubulin. **(A)** Expressions of Ak4, AMPK, and p-AMPK were analyzed by Western blotting (*n* = 5). **(B)** Expressions of Ak4, p-AMPK, and iNOS in A-769662 and AMPKα1/2 siRNA-treated Ak4 shRNA-treated M1 cells were analyzed by Western blotting (*n* = 4). **(C)** Production of IL-1β, IL-6, and TNFα (*n* = 4) were analysis by ELISA. *p < 0.05; **p < 0.01; ***p < 0.001 determined by unpaired, two-tailed Student's *t*-test **(A)** or one-way ANOVA **(B,C)** with mean ± SD.

Previous study has shown that AMPK activation is enhanced in A549 cell line by Ak4 shRNA treatment (19). Consistently, we found the level of p-AMPK was increased in Ak4 shRNA-treated M1 cells (Figure 4A). Treatment with A-769662 and metformin, two AMPK agonists, further reduced the expression of iNOS, IL-1β, IL-6, and TNF-α in Ak4 shRNA-treated cells (Figures 4B,C; Supplementary Figures 5A,B). Reversely, AMPKα1/2 siRNA blunted the effect of Ak4 shRNA (Figures 4B,C). While Ak4 promotes inflammatory genes by inhibiting AMPK activation, our study shows that AMPK agonists increase the level of Ak4 and silencing AMPKα1/2 reduces Ak4 expression (Figure 4B; Supplementary Figure 5A).

### Ak4 Regulates Several Biological Processes but Not the Polarization of M1/M2 Cells

**Figure 5:**
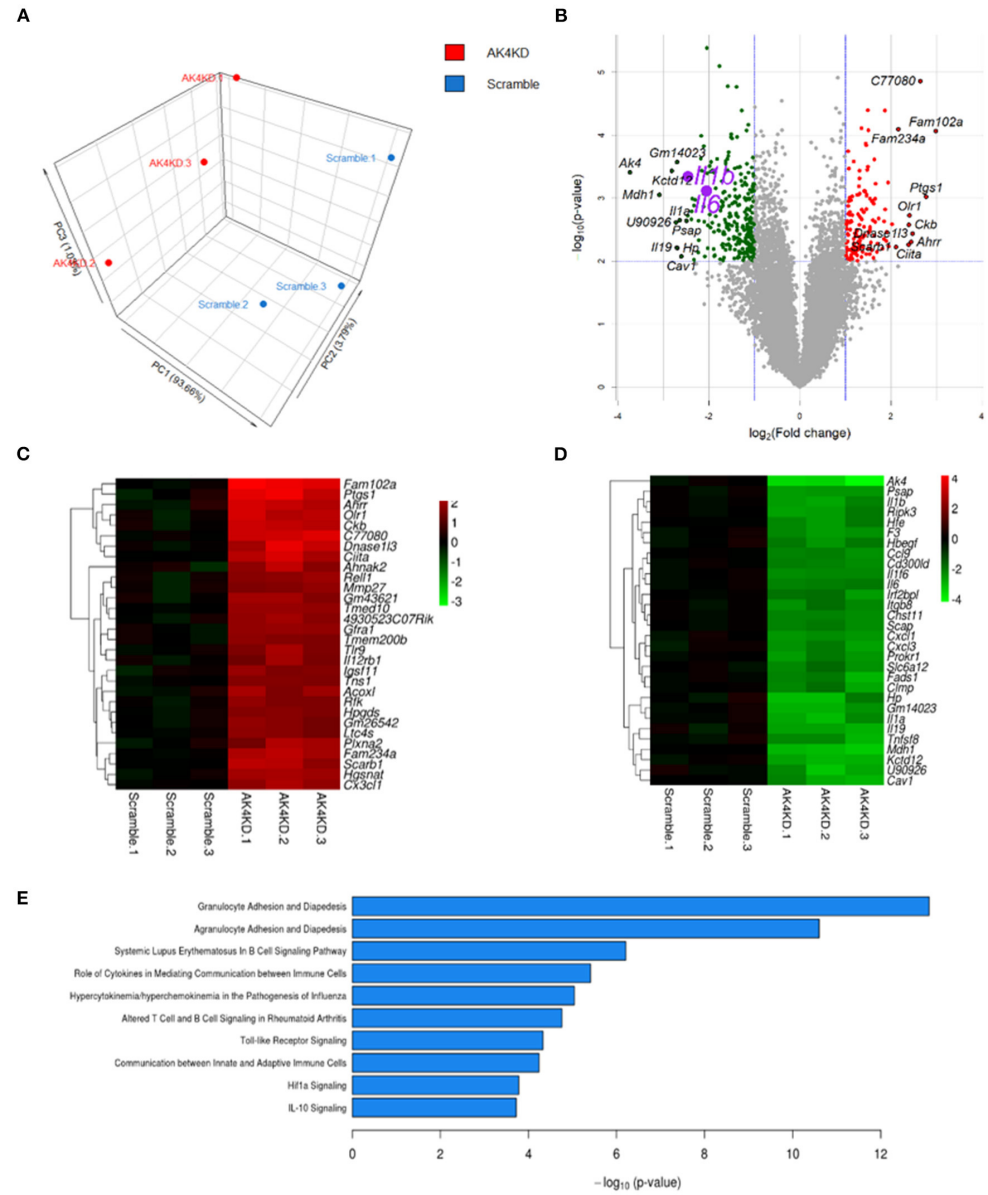
Ak4-regulated pathways in Ak4 shRNA-treated M1 cells were revealed by next generation sequencing. **(A)** Principal component analysis (PCA) of Ak4-regulated genes. PCA was plotted using expression of differentially expressed genes after Trimmed mean of M-values (TMM) normalization. Each dot represents one sample. Three independent experiments were performed for both Ak4 shRNA group (red) and scramble control (blue). **(B)** Volcano plots of differentially expressed genes in Ak4 shRNA-treated M1 macrophages. Criteria for selecting Ak4-regulated genes: fold change >2X or <1/2X and *P* < 0.001. Red points: up-regulated genes in Ak4 shRNA-treated M1 macrophages; green points: down-regulated genes in Ak4 shRNA-treated M1 macrophages. **(C)** Heat map and hierarchical cluster analysis of top 30 up-regulated genes in Ak4 shRNA-treated M1 macrophages. Red: up-regulated genes as compared to scramble shRNA control. **(D)** Heat map and hierarchical cluster analysis of top 30 down-regulated genes in Ak4 shRNA-treated M1 macrophages. Green: down-regulated genes as compared to scramble shRNA control. **(E)** Canonical pathways of Ak4-regulated genes using Ingenuity Pathway Analysis (IPA).

**Figure 6:**
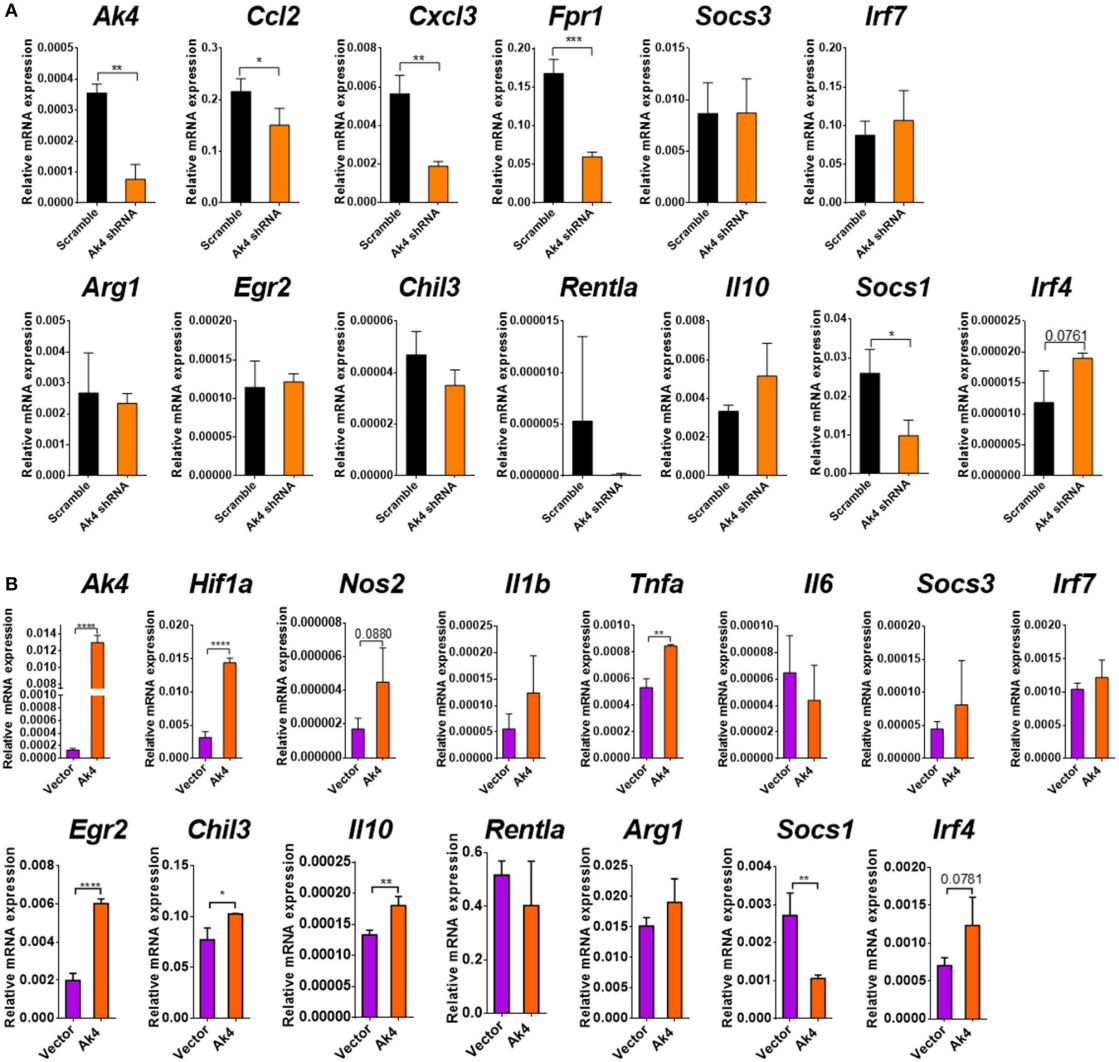
Ak4 does not regulate the polarization of M1/M2 cells. **(A)** Transcripts of *Ak4, Ccl2, Cxcl3, Fpr1, Arg1, Egr2, Chil3, Retnla, Il10, Socs1, Socs3, Irf4*, and *Irf7* in scramble shRNA- and Ak4 shRNA-treated M1 cells were analyzed by qPCR (*n* = 3). Gene expressions were compared to scramble shRNA control and normalized against *Actb*. **(B)** Transcripts of *Ak4, Hif1a, Nos2, Il1b, Il6, Tnfa, Il10, Egr2, Chil3, Retnla, Arg1, Socs3, Irf7, Socs1*, and *Irf4* in control (vector) and Ak4 overexpressing M2 cells were analyzed by qPCR (*n* = 3). Gene expressions were compared to control and normalized against *Actb*. *P < 0.05; **P < 0.01; ***P < 0.001; ****P < 0.0001 determined by unpaired, two-tailed Student's *t*-test with mean ± SD.

We subsequently examined the impact of Ak4 shRNA on the transcriptome of M1 macrophages. We stimulated the Ak4 shRNA- or scramble shRNA-transduced BMDMs with LPS and IFN-γ for 6 h. Total mRNAs were prepared from transduced cells and subjected to RNA-Seq. The Ak4 shRNA-transduced cells from thee independent experiments were transcriptomically segregated from scrambled shRNA-transduced cells in principal component analysis (PCA) (Figure 5A). Using a cut-off *p*-value of 0.01, fold-change (FC) > 2 or <1/2, we identified 145 genes, whose expression was enhanced, and 268 genes, whose expression was down-regulated by Ak4 shRNA treatment (Figures 5B–D). Interestingly, *Il1b* and *Il6* were among the top 10 most down-regulated genes by Ak4 shRNA (Figure 2B; Supplementary Tables 1, 2). Analyses of the differentially regulated genes (DRGs) with Ingenuity Pathway Analysis (IPA) showed that the DRGs participated in Hif1α signaling, a finding consistent with the data shown in Figures 2, 3, adhesion and diapedesis, cytokine-mediated communication, and TLR signaling (Figure 5E; Table 2). However, Gene Set Enrichment Analysis (GSEA) analysis of the RNA-seq data failed to detect any significant change in the expression of genes regulating M1/M2 polarization (Supplementary Figures 6A,B). Subsequent qPCR analyses also demonstrated that Ak4 shRNA treatment of M1 cells had no consistent effect on the expression of M1 genes, such as *Scos3 and Irf7*, and M2 genes, such as *Arg1, Egr2, Chil3, Rentla, Il10, Socs1*, and *Irf4* (Figure 6A). Moreover, Ak4 overexpression in M2 cells had no consistent effect on the expression of M1 genes, such as *Nos2, Il6, Socs3*, and *Irf7*, and M2 genes, such as *Egr2, Chil3, Il10, Rentla, Arg1*, and *Irf4* (Figure 6B).

**Table 2:** Top 10 Ak4-regulated canonical pathways and genes involved were listed.

**Ingenuity canonical pathways**	**Genes**
Granulocyte adhesion and diapedesis	*Ccl2, Ccl6, Ccl7, Ccl9, Csf3, Cx3cl1, Cxcl14, Cxcl2, Cxcl3, Cxcl3, Fpr1, Il1a, Il1b*,
	*Il36a, Il36g, Itga6, Mmp10, Mmp12, Mmp13, Mmp19, Mmp27, Pf4*
Agranulocyte adhesion and diapedesis	*Ccl2, Ccl6, Ccl7, Ccl9, Cx3cl1, Cxcl14, Cxcl2, Cxcl3, Cxcl3, Il1a, Il1b, Il36a, Il36g*,
	*Itga6, Mmp10, Mmp12, Mmp13, Mmp19, Mmp27, Pf4*
Systemic lupus erythematosus in B cell signaling pathway	*Bcl2l11, Btk, Fgr, Gab1, Ifnb1, Il1a, Il1b, Il36a, Il36g, Il6, Inpp5b, Irak1*,
	*Map3k14, Nfatc1, Tlr9, Tnfsf15, Tnfsf8, Traf3*
Role of cytokines in mediating communication between immune cells	*Csf3, Ifnb1, Il1a, Il1b, Il36a, Il36g, Il6*
Role of hypercytokinemia/hyperchemokinemia in the pathogenesis of influenza	*Ifnb1, Il1a, Il1b, Il36a, Il36g, Il6*
Altered T cell and B cell signaling in rheumatoid arthritis	*Il1a, Il1b, Il36a, Il36g, Il6, Map3k14, Spp1, Tlr9, Traf3*
Toll-like receptor signaling	*Il1a, Il1b, Il36a, Il36g, Irak1, Map3k14, Mapk14, Tlr9*
Communication between innate and adaptive immune cells	*Ccl9, Ifnb1, Il1a, Il1b, Il36a, Il36g, Il6, Tlr9*
HIF1α signaling	*Apex1, Edn1, Mapk14, Mmp10, Mmp12, Mmp13, Mmp19, Mmp27, Slc2a1*
IL-10 signaling	*Il1a, Il1b, Il36a, Il36g, Il6, Map3k14, Mapk14*

*Ak4-regulated canonical pathways and genes were identified by Ingenuity Pathway Analysis (IPA)*.

In conclusion, iNOS, Nox2, and pro-inflammatory cytokine, IL-1β, IL-6, and TNF-α expressions are regulated by Ak4-Hif1α and Ak4-AMPK bidirectional loops. Moreover, Ak4 mediates several pathways important for M1 function. However, Ak4 does not regulate M1/M2 polarization.

## Discussion

We demonstrated in this study that Ak4 positively regulates the expression of inflammation genes, such as IL-1β, IL-6, TNF-α, Nox2, and iNOS, in M1 cells through at least two mechanisms, one, by forming a positive feedback loop with Hif1α, and, second by inhibiting the activation of AMPK. A previous study showed in non-small cell lung cancer cells (NSCLC) that Ak4 stabilizes Hif1α protein through inhibition of prolyl hydroxylase (PHD) (22), our data suggest that Ak4 not only stabilizes Hif1α protein but also enhances its transcription (Figure 3C; Supplementary Figures 3D,H). Consistent with published data showing that Ak4 gene is a target of Hif1α (20, 43) and that Ak4 transcript is reduced in Hif1α-deficient hematopoietic cells (44), our study also suggests that Ak4 and Hif1α form a positive feedback loop in M1 cells (Figures 3A–C). However, how Ak4 regulates the transcription of Hif1α is still unclear. We propose the following scenario based on the results of our study revealed in Ak4 silenced M1 cells: First, Ak4 regulates ATP level and ADP/ATP ratio (Figure 1E), suggesting Hif1α transcription may be mediated by the concentration of ATP or ADP/ATP ratio. Second, Ak4 controls ROS production (Figure 1F), which can subsequently activate NF-κB, a key transcription factor of Hif1α (45–47). Thus, Hif1α transcription may be positively regulated by Ak4-ROS-NF-κB pathway. Third, Ak4 inhibits AMPK activation (Figure 4; Supplementary Figure 5). Activated AMPK inhibits NF-κB activity, leading to reduced Hif1α transcription (46–48). It is thus likely that Hif1α transcription may also be regulated through Ak4-AMPK-NF-κB axis. Our results also showed that Ak4 promotes the expression of inflammatory genes by inhibiting AMPK activation (Figure 4). AMPK agonists increase the level of Ak4 and silencing AMPKα1/2 reduces Ak4 expression (Figure 4B; Supplementary Figure 5A). Based on our results together with published data, we propose that Ak4 through promoting Hif1a transcription and inhibiting AMPK activation positively regulates inflammation gene expression.

Ak4 is located in mitochondria matrix that maintains ATP/ADP/AMP homeostasis (16, 17). However, whether Ak4 affects mitochondria function in macrophage is still unclear. Previous study showed that glycolysis is enhanced in Ak4-overexpressing CL1-0 cells (22). Oxygen consumption rate (OCR) is enhanced in Ak4-silenced Hela cells (19). The metabolic state of M1 macrophages preferentially uses glycolysis over oxidative phosphorylation (OXPHOS) for the source of energy (31). Our results showed that glycolysis, but not OXPHOS or OCR, is impaired in Ak4-silenced M1 cells (Figure 1H). It is reported that mitochondria mass and metabolites are both altered in Ak4 shRNA-silenced or Ak4-overexpressing Hela cells (19). In addition, our data show that Ak4 suppresses AMPK activation which is known to mediate mitochondria biogenesis, OXPHOS and ATP production (49). Based on these observations, it is our speculation that Ak4 contributes to the function of mitochondria in M1 macrophages.

Our study reveals a potential mechanism linking energy metabolism and inflammation in macrophages. The key question for this study is why Ak4 up-regulation is so critical for macrophage inflammation. Ak4 may be involved in the following possible ways. First, the catalytic function of Ak4 in the reversible reaction of ADP production through the consumption of ATP and AMP indeed affects mitochondrial cues necessary for macrophage inflammation. Second, Ak4-mediated consumption of AMP may suppress AMPK activity, thereby affecting transcriptional control of specific M1 gene signature and inflammatory cytokine production. Third, Ak4 may regulate possible mechanisms mentioned above, thereby altering Hif-1α signal in transcriptional programming specified for M1 function. Moreover, our RNA-seq analyses show that in addition to regulation of inflammation-related genes in M1 cells, Ak4 also regulates biological processes, such as migration, TLR signaling and communication between innate and adaptive cells. Our work raises the possibility that Ak4 can be a potential target in treating M1-mediated inflammation.

## Data Availability Statement

The datasets presented in this study can be found in online repositories. The names of the repository/repositories and accession number(s) can be found in the article/Supplementary Materials.

## Ethics Statement

The animal study was reviewed and approved by Institutional Animal Care and Use Committee of National Taiwan University College of Medicine.

## Author Contributions

S-CM: conceptualization, supervision, and writing—review and editing. W-YC, C-YH, and S-CM: data curation and validation. C-YH, TWC, L-CL, and S-CM: formal analysis. W-YC and S-CM: funding acquisition, project administration, and writing—original draft. C-YH, Q-YY, L-CL, and S-CM: investigation. W-YC, C-YH, and TWC: methodology. All authors: contributed to the article and approved the submitted version.

## Conflict of Interest

The authors declare that the research was conducted in the absence of any commercial or financial relationships that could be construed as a potential conflict of interest.

## Supplementary Material

Supplementary Figure 1 The definition of M1/M2 marker. **(A)** Expressions of CD86 and CD206 were analyzed by flow cytometry (*n* = 3). **(B)** The mRNA expression of *Nos2, Tnfa, Arg1*, and *Egr2* were analyzed by qPCR in M0, M1, and M2 (*n* = 3). Relative gene expressions were normalized against *Actb*. ^**^*P* < 0.01; ^***^*P* < 0.001; ^****^*P* < 0.00001 determined by unpaired *T*-test, two tailed with mean ± SD.

Supplementary Figure 2 Ak4 protein is located in the mitochondria of M1 macrophages. LPS/IFN-γ stimulated BMDMs (5 × 10^7^) were lysed with extraction buffer. Expressions of Ak4, COX IV (mitochondria internal control), and β-Actin (cytosol internal control) proteins were analyzed by Western blotting.

Supplementary Figure 3 Expressions of inflammation-related mRNA in Ak4-silenced M1 macrophages. Transcripts of pro-inflammatory cytokine *Il1b, Il6, Tnfa, Nos2*, and *Hif1a* genes in Ak4-silenced and scramble control M1 macrophages were analyzed by real-time PCR analysis. Transcripts of *Il1b, Il6*, and *Tnfa* were analyzed for 2 h M1-polarized cells. Transcripts of *Ak4, Nos2*, and *Hif1a* were analyzed for 24 h M1-polarized cells. Relative gene expressions were normalized against *Actb*. **(A)** The amount of ATP and ADP/ATP from BMDMs treated with Ak4 siRNA were measured (*n* = 5). **(B–E)** Relative mRNA expression of **(B)** *Ak4* (n = 3), **(C)** *Il1b* (*n* = 7), *Il6* (*n* = 3), *Tnfa* (*n* = 5), **(D)** *Hif1a* (*n* = 5), and **(E)** *Nos2* (*n* = 5) in Ak4 shRNA- and scramble shRNA-treated M1 macrophages. **(F–H)** Relative mRNA expression of **(F)** *Ak4*, **(G)** *Il1b, Il6, Tnfa*, **(H)** *Hif1a*, and *Nos2* in Ak4 siRNA- and scramble siRNA-treated M1 cells (*n* = 3). **(I,J)** Relative mRNA expressions **(I)** and protein production **(J)** of *Ak4, Il1b, Il6*, and *Tnfa* in Ak4 siRNA- and scramble siRNA-treated peritoneal M1 macrophages (*n* = 5). ^*^*P* < 0.05; ^**^*P* < 0.01; ^***^*P* < 0.001; ^****^*P* < 0.0001 determined by unpaired T-test, two tailed with mean ± SD.

Supplementary Figure 4 Pro-inflammatory cytokines and iNOS were positively regulated by Hif1α. Expressions of pro-inflammatory cytokines IL-1β, IL-6, TNF-α, Hif1α, iNOS, and Ak4 protein in Hif1α-silenced and scramble control M1 macrophages or 0.5 mM DMOG-treated M1 macrophages were analyzed by ELISA or Western blotting analysis. Relative protein expressions were normalized against α-Tubulin. **(A)** Hif1α protein expressions were analyzed by Western blotting (*n* = 3). **(B)** Production of cytokines IL-1β (*n* = 3), IL-6 (*n* = 9), and TNF-α (*n* = 9) were measured by ELISA. **(C)** Relative protein expressions of iNOS and Ak4 in Hif1α KD and scramble M1 macrophages (*n* = 3). **(D)** overexpression of HIF1α protein in DMOG-treated M1 macrophages were analyzed by Western blotting (*n* = 8). **(E)** Production of cytokines IL-1β, IL-6, and TNF-α were measured by ELISA (*n* = 3). **(F)** Relative protein expressions of iNOS and Ak4 in DMOD-treated M1 macrophages (*n* = 8). ^*^*P* < 0.05; ^**^*P* < 0.01 determined by unpaired T-test, two tailed with mean ± SD.

Supplementary Figure 5 Ak4 inhibits the activation of AMPK to promote inflammation gene expressions in M1 macrophages. Expressions of Ak4, p-AMPK, AMPK in scramble shRNA- and Ak4 shRNA-treated M1 macrophages were analyzed by Western blotting. Relative protein expressions were normalized against β-actin. **(A)** Expressions of Ak4, p-AMPK, and iNOS in metformin-treated Ak4 shRNA M1 cells were analyzed by Western blotting (*n* = 4). **(B)** Production of IL-1β (*n* = 7), IL-6 (*n* = 7), and TNFα (*n* = 4) were analyzed by ELISA. ^*^*p* < 0.05; ^**^*p* < 0.01; ^***^*p* < 0.001 determined by one-way ANOVA with mean ± SD.

Supplementary Figure 6 Comparison of Ak4 siRNA-treated M1 gene profile to M1/M2 activated genes set by GSEA analysis. Ak4 siRNA- and scramble siRNA-treated M1 cells. **(A,B)** Gene set enrichment analysis (GSEA) pathway enrichment plots indicating the representative gene sets, classically activated up/down-regulated genes **(A)** and alternatively activated up/down-regulated genes **(B)**, enriched in Ak4 shRNA-treated M1 cells versus scramble-treated M1 cells. GSE69607 gene set were used for GSEA analysis. Normalized enrichment score (NES) and *P* values are shown for each gene set. *P* values were calculated by Kolmogorov-Smirnov test.

Supplementary Table 1 Top 30 up-regulated genes in Ak4 shRNA-treated M1 cells were listed. Up-regulated genes in Ak4 shRNA-treated M1 cells were identified by IPA. The full names and functions of genes were searched on GeneCards (https://www.genecards.org/).

Supplementary Table 2 Top 30 down-regulated genes in Ak4 shRNA-treated M1 cells were listed. Down-regulated genes in Ak4 shRNA-treated M1 cells were identified by IPA. The full names and functions of genes were searched on GeneCards (https://www.genecards.org/).
