# Supplementary figures and images for: Adenylate Kinase 4 Promotes Inflammatory Gene Expression via Hif1α and AMPK in Macrophages
Source: Front Immunol. 2021 Mar 15;12:630318. doi: 10.3389/fimmu.2021.630318 (PMC8005550; doi:10.3389/fimmu.2021.630318)

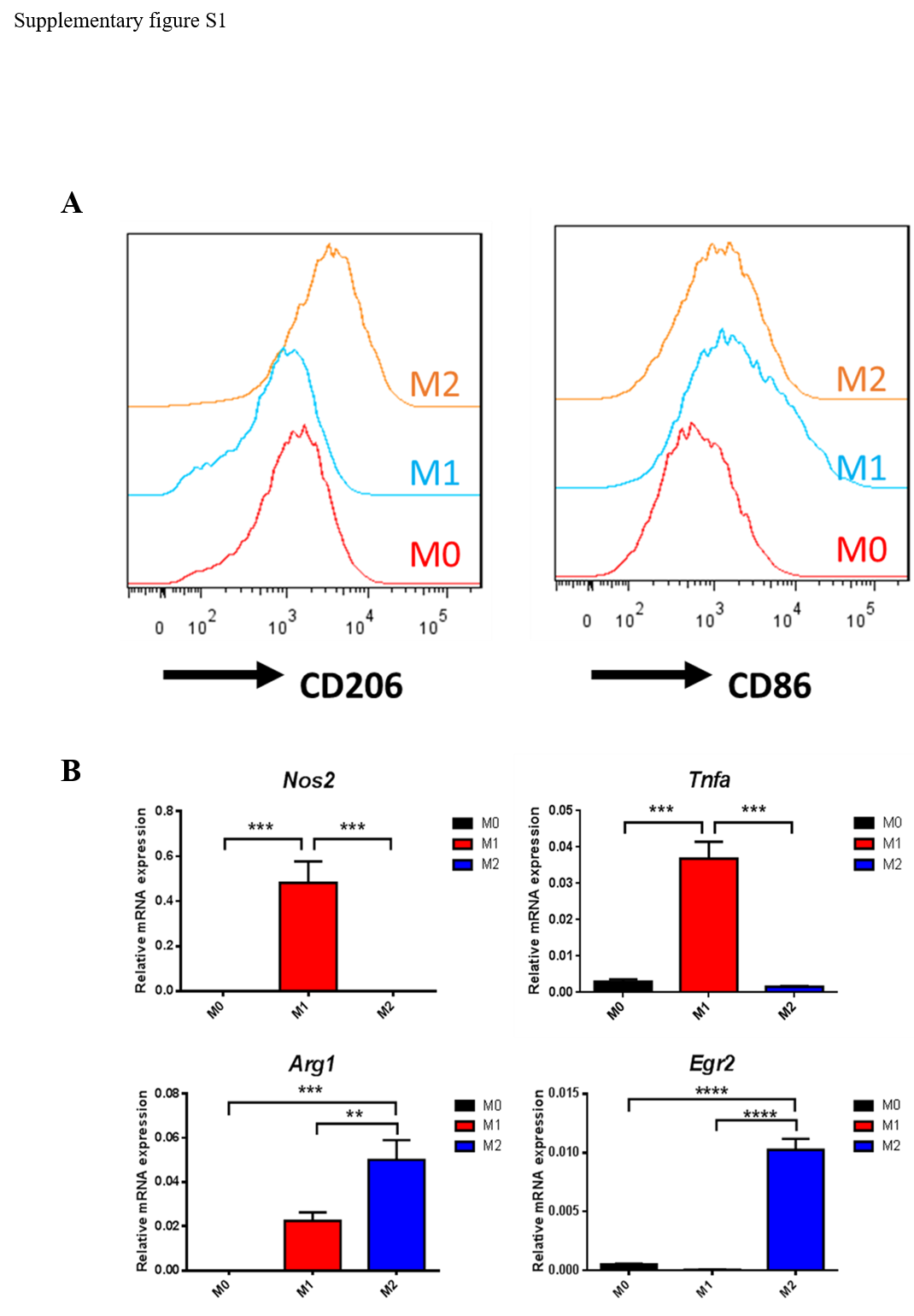

Supplement: Supplementary Figure 1 — The definition of M1/M2 marker. (A) Expressions of CD86 and CD206 were analyzed by flow cytometry (n = 3). (B) The mRNA expression of Nos2, Tnfa, Arg1, and Egr2 were analyzed by qPCR in M0, M1, and M2 (n = 3). Relative gene expressions were normalized against Actb. **P < 0.01; ***P < 0.001; ****P < 0.00001 determined by unpaired T-test, two tailed with mean ± SD. [file Image_1.TIF]

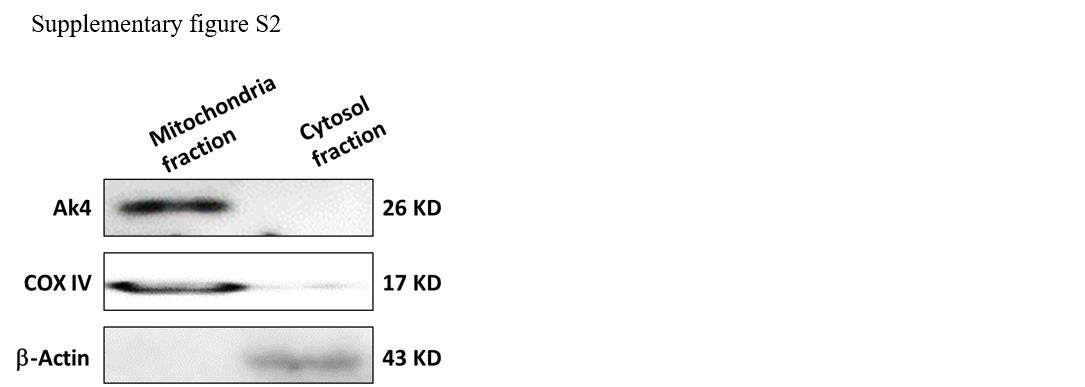

Supplement: Supplementary Figure 2 — Ak4 protein is located in the mitochondria of M1 macrophages. LPS/IFN-γ stimulated BMDMs (5 × 10^7^) were lysed with extraction buffer. Expressions of Ak4, COX IV (mitochondria internal control), and β-Actin (cytosol internal control) proteins were analyzed by Western blotting. [file Image_2.TIF]

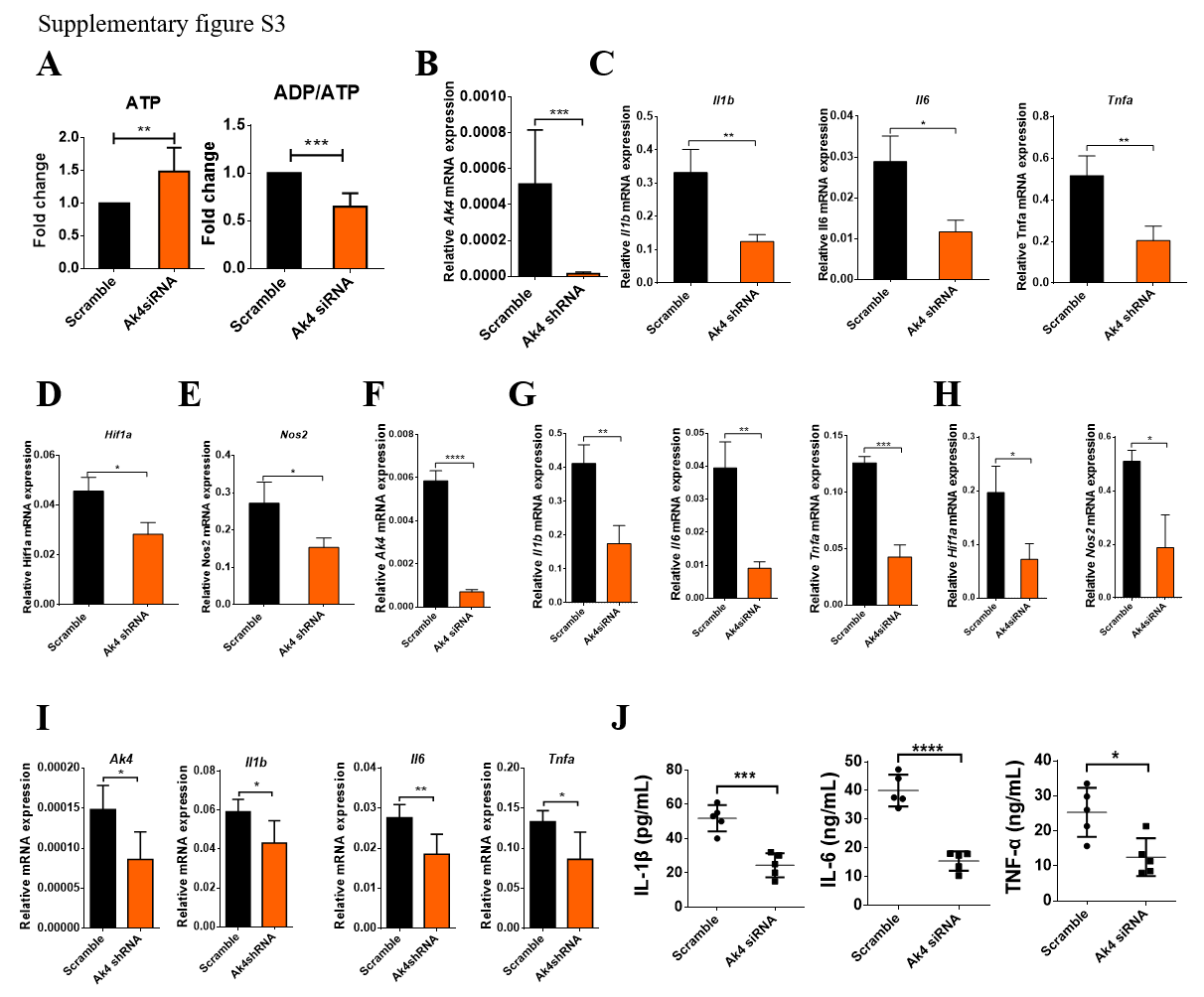

Supplement: Supplementary Figure 3 — Expressions of inflammation-related mRNA in Ak4-silenced M1 macrophages. Transcripts of pro-inflammatory cytokine Il1b, Il6, Tnfa, Nos2, and Hif1a genes in Ak4-silenced and scramble control M1 macrophages were analyzed by real-time PCR analysis. Transcripts of Il1b, Il6, and Tnfa were analyzed for 2 h M1-polarized cells. Transcripts of Ak4, Nos2, and Hif1a were analyzed for 24 h M1-polarized cells. Relative gene expressions were normalized against Actb. (A) The amount of ATP and ADP/ATP from BMDMs treated with Ak4 siRNA were measured (n = 5). (B–E) Relative mRNA expression of (B) Ak4 (n = 3), (C) Il1b (n = 7), Il6 (n = 3), Tnfa (n = 5), (D) Hif1a (n = 5), and (E) Nos2 (n = 5) in Ak4 shRNA- and scramble shRNA-treated M1 macrophages. (F–H) Relative mRNA expression of (F) Ak4, (G) Il1b, Il6, Tnfa, (H) Hif1a, and Nos2 in Ak4 siRNA- and scramble siRNA-treated M1 cells (n = 3). (I,J) Relative mRNA expressions (I) and protein production (J) of Ak4, Il1b, Il6, and Tnfa in Ak4 siRNA- and scramble siRNA-treated peritoneal M1 macrophages (n = 5). *P < 0.05; **P < 0.01; ***P < 0.001; ****P < 0.0001 determined by unpaired T-test, two tailed with mean ± SD. [file Image_3.TIF]

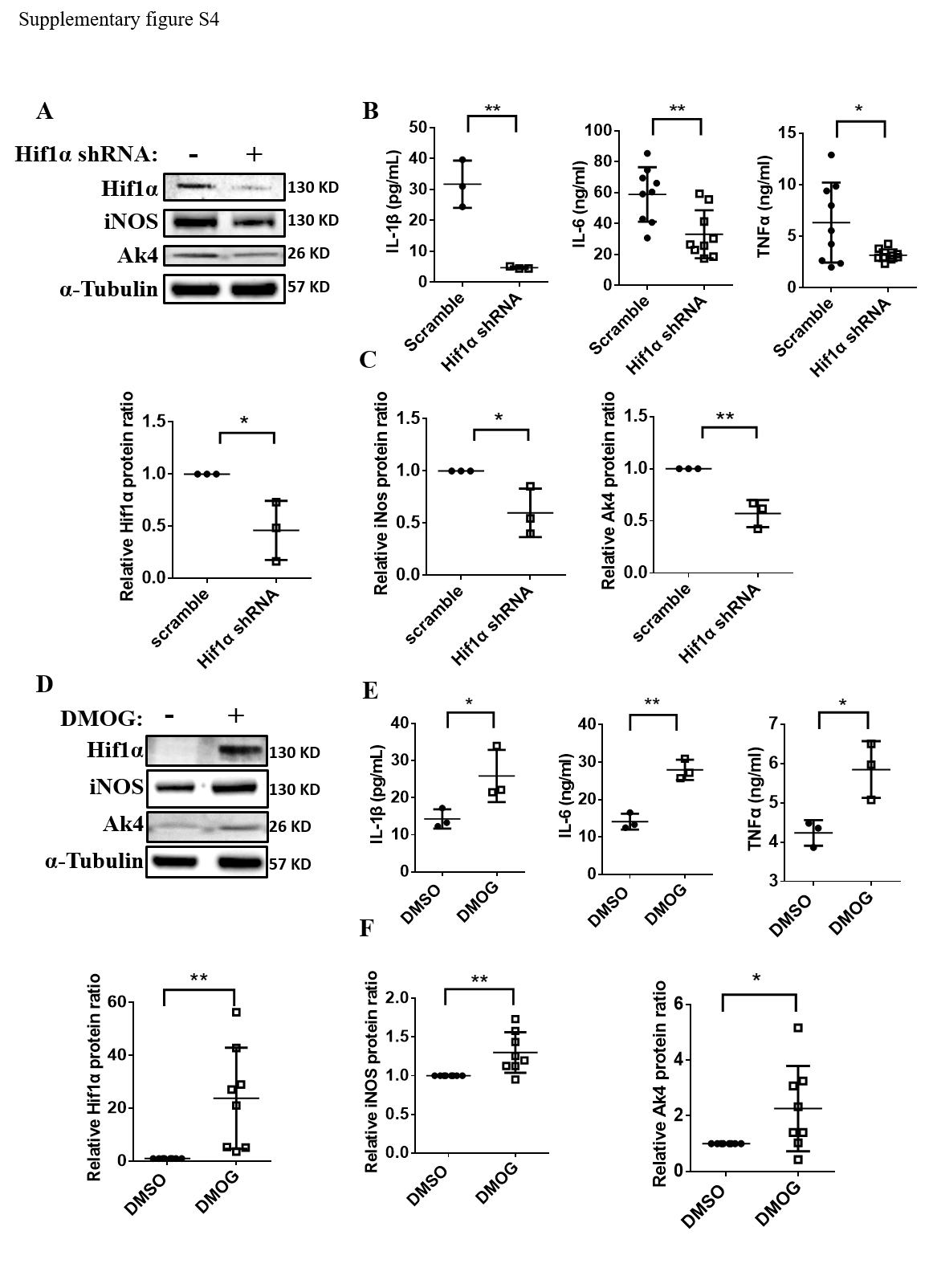

Supplement: Supplementary Figure 4 — Pro-inflammatory cytokines and iNOS were positively regulated by Hif1α. Expressions of pro-inflammatory cytokines IL-1β, IL-6, TNF-α, Hif1α, iNOS, and Ak4 protein in Hif1α-silenced and scramble control M1 macrophages or 0.5 mM DMOG-treated M1 macrophages were analyzed by ELISA or Western blotting analysis. Relative protein expressions were normalized against α-Tubulin. (A) Hif1α protein expressions were analyzed by Western blotting (n = 3). (B) Production of cytokines IL-1β (n = 3), IL-6 (n = 9), and TNF-α (n = 9) were measured by ELISA. (C) Relative protein expressions of iNOS and Ak4 in Hif1α KD and scramble M1 macrophages (n = 3). (D) overexpression of HIF1α protein in DMOG-treated M1 macrophages were analyzed by Western blotting (n = 8). (E) Production of cytokines IL-1β, IL-6, and TNF-α were measured by ELISA (n = 3). (F) Relative protein expressions of iNOS and Ak4 in DMOD-treated M1 macrophages (n = 8). *P < 0.05; **P < 0.01 determined by unpaired T-test, two tailed with mean ± SD. [file Image_4.TIF]

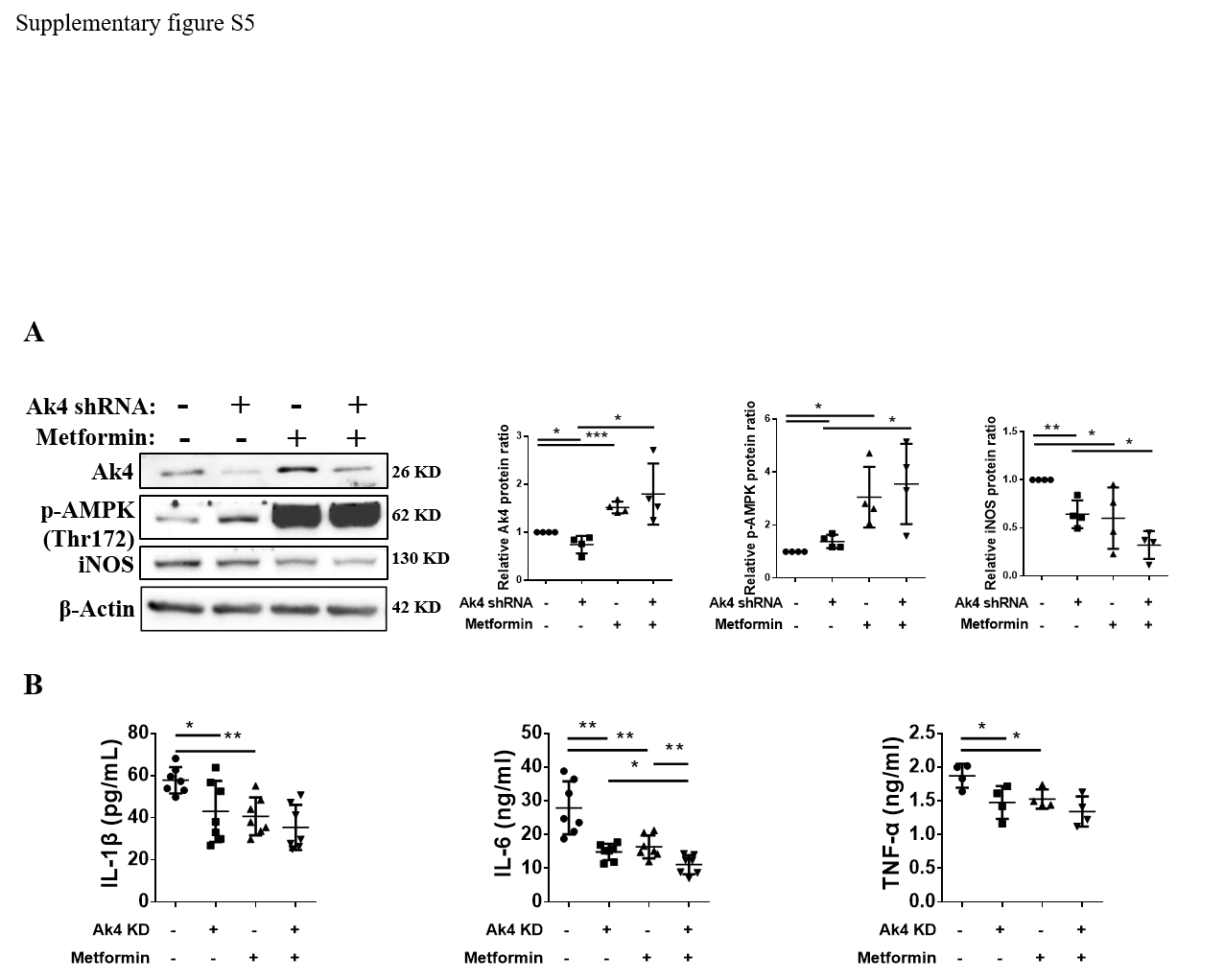

Supplement: Supplementary Figure 5 — Ak4 inhibits the activation of AMPK to promote inflammation gene expressions in M1 macrophages. Expressions of Ak4, p-AMPK, AMPK in scramble shRNA- and Ak4 shRNA-treated M1 macrophages were analyzed by Western blotting. Relative protein expressions were normalized against β-actin. (A) Expressions of Ak4, p-AMPK, and iNOS in metformin-treated Ak4 shRNA M1 cells were analyzed by Western blotting (n = 4). (B) Production of IL-1β (n = 7), IL-6 (n = 7), and TNFα (n = 4) were analyzed by ELISA. *p < 0.05; **p < 0.01; ***p < 0.001 determined by one-way ANOVA with mean ± SD. [file Image_5.TIF]

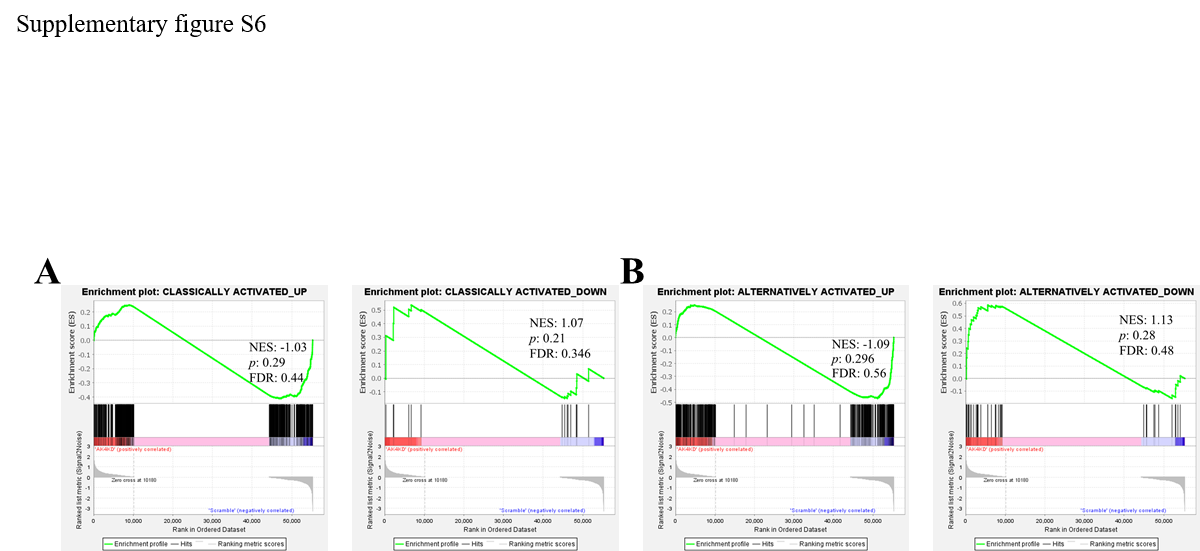

Supplement: Supplementary Figure 6 — Comparison of Ak4 siRNA-treated M1 gene profile to M1/M2 activated genes set by GSEA analysis. Ak4 siRNA- and scramble siRNA-treated M1 cells. (A,B) Gene set enrichment analysis (GSEA) pathway enrichment plots indicating the representative gene sets, classically activated up/down-regulated genes (A) and alternatively activated up/down-regulated genes (B), enriched in Ak4 shRNA-treated M1 cells versus scramble-treated M1 cells. GSE69607 gene set were used for GSEA analysis. Normalized enrichment score (NES) and P values are shown for each gene set. P values were calculated by Kolmogorov-Smirnov test. [file Image_6.TIF]

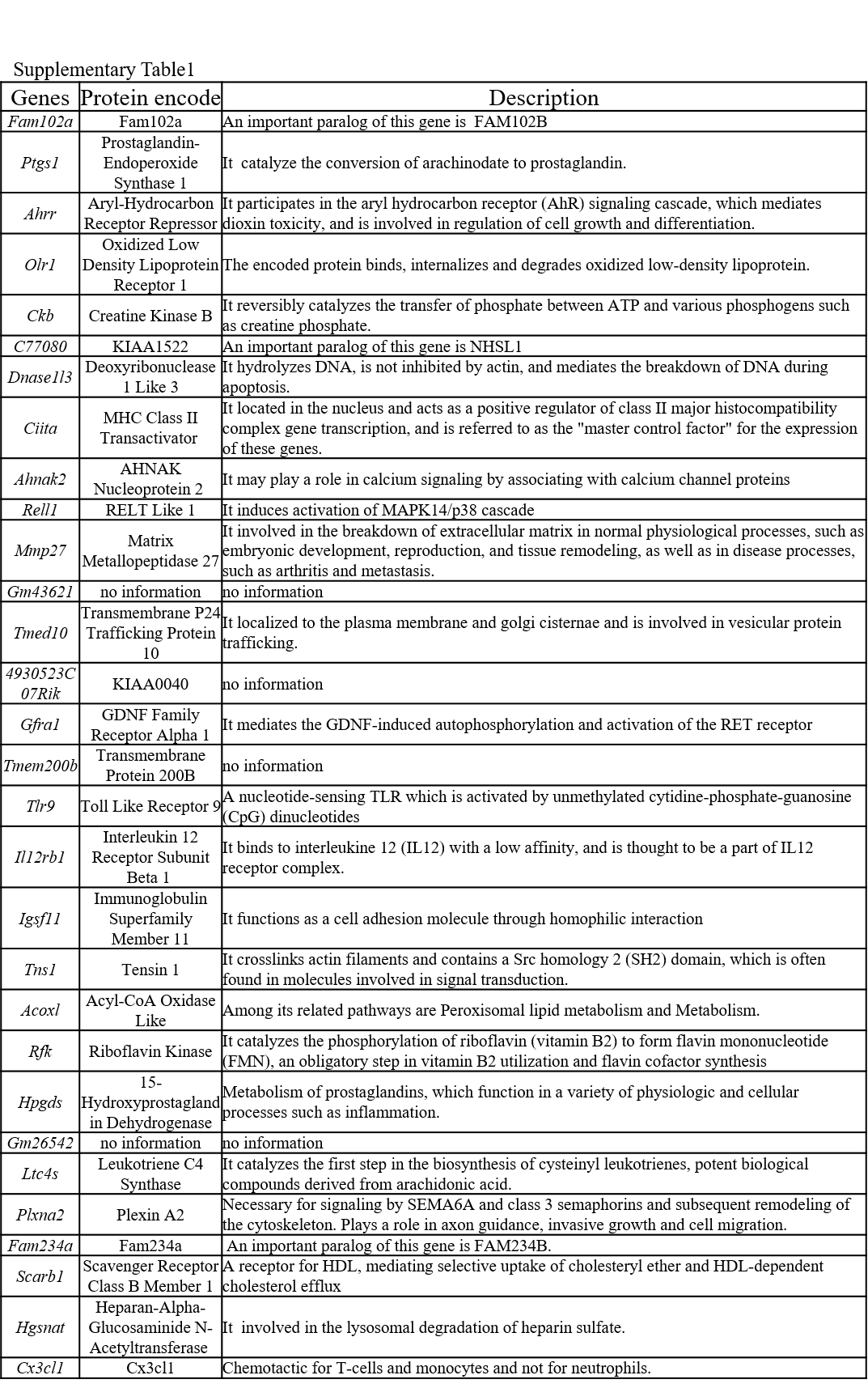

Supplement: Supplementary Table 1 — Top 30 up-regulated genes in Ak4 shRNA-treated M1 cells were listed. Up-regulated genes in Ak4 shRNA-treated M1 cells were identified by IPA. The full names and functions of genes were searched on GeneCards (https://www.genecards.org/). [file Image_7.TIF]

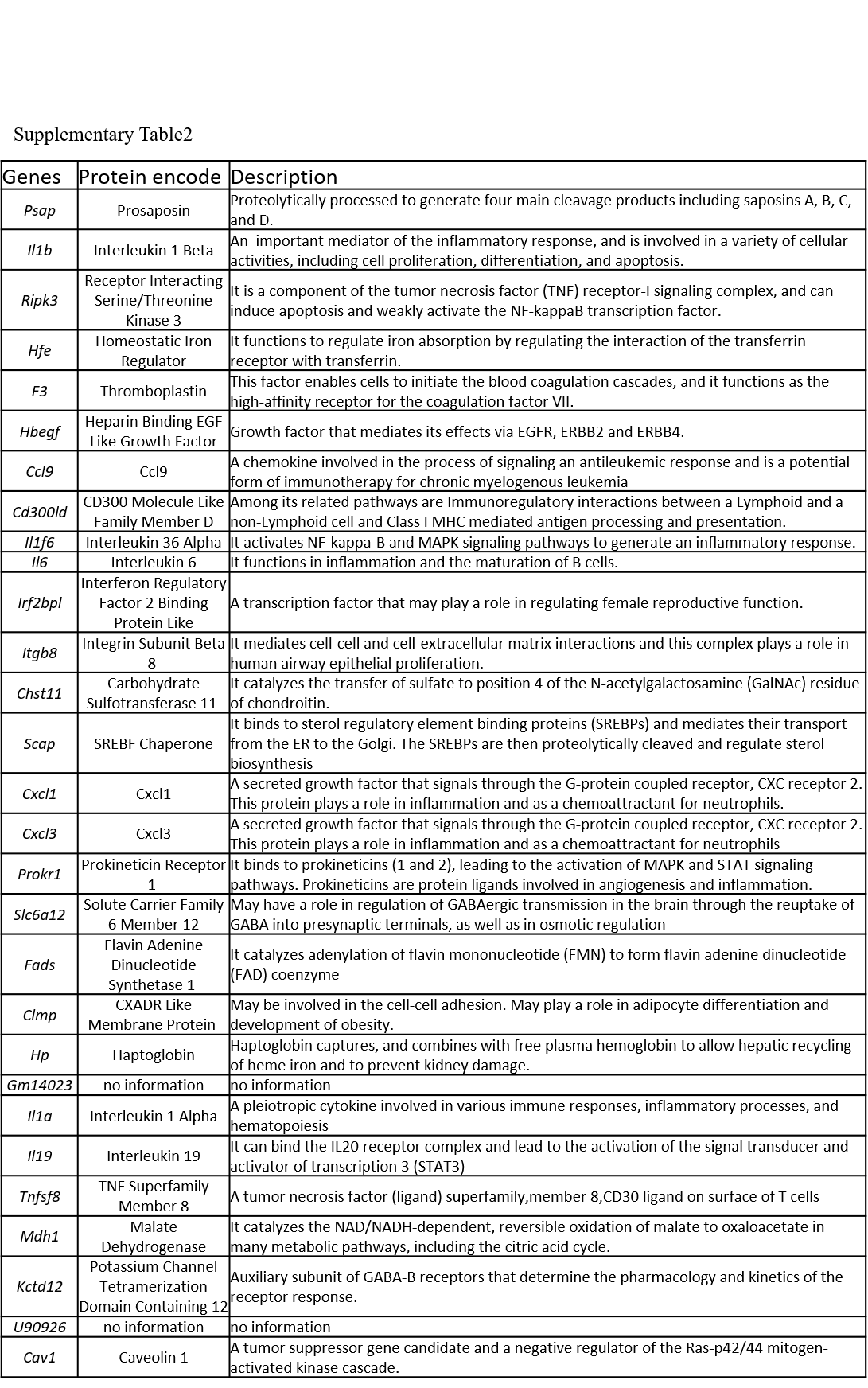

Supplement: Supplementary Table 2 — Top 30 down-regulated genes in Ak4 shRNA-treated M1 cells were listed. Down-regulated genes in Ak4 shRNA-treated M1 cells were identified by IPA. The full names and functions of genes were searched on GeneCards (https://www.genecards.org/). [file Image_8.TIF]
